# Genetic Characterization and Pathogenicity of a Recombinant Porcine Reproductive and Respiratory Syndrome Virus Strain in China

**DOI:** 10.3390/v16060993

**Published:** 2024-06-20

**Authors:** Yan Ouyang, Yingbing Du, Hejin Zhang, Jiahui Guo, Zheng Sun, Xiuxin Luo, Xiaowei Mei, Shaobo Xiao, Liurong Fang, Yanrong Zhou

**Affiliations:** 1 National Key Laboratory of Agricultural Microbiology, College of Veterinary Medicine, Huazhong Agricultural University, Wuhan 430070, China; goldmanxxc@126.com (Y.O.); dyblky@webmail.hzau.edu.cn (Y.D.); zhi1993@webmail.hzau.edu.cn (H.Z.); guojiahui@webmail.hzau.edu.cn (J.G.); pullsticker@webmail.hzau.edu.cn (Z.S.); xiuxinluo@163.com (X.L.); meixiaowei198377@163.com (X.M.); vet@mail.hzau.edu.cn (S.X.); fanglr@mail.hzau.edu.cn (L.F.); 2 College of Agriculture, Hubei Three Gorges Polytechnic, Yichang 443000, China; 3 The Key Laboratory of Preventive Veterinary Medicine in Hubei Province, Cooperative Innovation Center for Sustainable Pig Production, Wuhan 430070, China

**Keywords:** PRRSV, NADC30-like, amino acid deletion, recombination, pathogenicity

## Abstract

Since it was first reported in 2013, the NADC30-like PRRSV has been epidemic in China. Hubei Province is known as China’s key hog-exporting region. To understand the prevalence and genetic variation of PRRSV, herein, we detected and analyzed 317 lung tissue samples from pigs with respiratory disease in Hubei Province, and demonstrated that the NADC30-like strain was the second-most predominant strain during 2017–2018, following the highly pathogenic PRRSV (HP-PRRSV). Additionally, we isolated a new NADC30-like PRRSV strain, named CHN-HB-2018, which could be stably passaged in Marc-145 cells. Genetic characterization analysis showed that compared with the NADC30 strain, the CHN-HB-2018 strain had several amino acid variations in glycoprotein (GP) 3, GP5, and nonstructural protein 2 (NSP2). Moreover, the CHN-HB-2018 strain showed a unique 5-amino acid (aa) deletion in NSP2, which has not previously been reported. Gene recombination analysis identified the CHN-HB-2018 strain as a potentially recombinant PRRSV of the NADC30-like strain and HP-PRRSV. Animal experiments indicated that the CHN-HB-2018 strain has a mild pathogenicity, with no mortality and only mild fever observed in piglets. This study contributes to defining the evolutionary characteristics of PRRSV and its molecular epidemiology in Hubei Province, and provides a potential candidate strain for PRRSV vaccine development.

## 1. Introduction

Porcine reproductive and respiratory syndrome virus (PRRSV) is the etiologic agent of PRRS, an economically harmful infectious disease that can cause respiratory distress in pigs of all ages and reproductive failure in sows [1,2,3,4]. PRRSV is a single-stranded, positive-sense RNA virus of the *Nidovirales* order, Arteriviridae family, Arterivirus genus, and its genome is ~15 kb long with no less than 11 open reading frames (ORFs) [5,6]. Studies have shown that polyproteins pp1a and pp1ab, encoded by ORF1a and ORF1b, can be further cleaved into 14 nonstructural proteins (NSPs), namely NSP1α, NSP1β, NSP2-6, NSP7α, NSP7β, and NSP8-12 [7,8,9]. Additionally, eight structural proteins and two NSPs are encoded by the remaining ORFs, which are envelope (E) protein, glycoprotein 2a (GP2a), GP3-5, ORF5a, membrane (M) protein, nucleocapsid (N) protein, NSP2N, and NSP2TF [10,11]. 

As an RNA virus, genetic diversity is fairly high for PRRSV, which can be classified into PRRSV-1 (Betaarterivirus suid 1) and PRRSV-2 (Betaarterivirus suid 2), with less than 60% identity at the whole-genome level [12]. Currently, the predominant PRRSV-2 strains in China are mainly co-existing Lineages of 8, 1, 3, and 5 [13]. Since 2006, the highly pathogenic PRRSV (HP-PRRSV) of Lineage 8 has been prevalent in China [14,15,16], and the year 2013 witnessed the emergence of the NADC30-like PRRSV strain (Lineage 1) in China [17], followed by its increasing prevalence in numerous pig farms throughout China, even taking the place of HP-PRRSV as the dominant PRRSV strain. With a unique discontinuous deletion of 131-aa in NSP2, NADC30-like strains have genetic similarity with the USA NADC30 strain, which was first identified in the USA in 2008 and was shown to possess weak pathogenicity [18]. Although less pathogenic than HP-PRRSV, NADC30-like PRRSV is highly susceptible to recombination with HP-PRRSV, classical PRRSV, or vaccine strains in China [19]. 

Recombination always occurs between different PRRSV lineages/sub-lineages, so it has been regarded as one of the most important mechanisms in PRRSV evolution, increasing viral genetic diversity coupled with unpredictable variance in viral pathogenicity. For example, Wang et al. reported that a JXA1 and NADC30 recombinant PRRSV strain exhibited lower pathogenicity than the JXA1 strain (HP-PRRSV), but higher pathogenicity than the NADC30 strain [20]. In recent years, due to the widespread use of live attenuated vaccines, there have been frequent reports about field strain and vaccine strain recombination events, which may account for virulence reversal of vaccine strains [21,22]. Collectively, a key immune evasion strategy for PRRSV is generating novel strains through high-frequency recombination, which has increased the difficulty of PRRS prevention and control [23,24,25]. 

PRRSV has been prevalent in China for over 20 years, undergoing continuous mutations [26]. Currently, more than 80% of pig farms in China are deeply affected by PRRS, making it one of the primary infectious diseases impeding the healthy growth of the pig-farming industry [11]. Therefore, it is crucial to consistently monitor the prevalence and genetic variations of PRRSV in concentrated pig-breeding areas in China. Hubei Province is one of the key hog-farming areas in China. This study aims to investigate the genetic characteristics of the current PRRSV strains circulating in Hubei Province, China, providing a foundation for the prevention and control of PRRSV.

## 2. Materials and Methods

### 2.1. Collection and RT-PCR Detection of PRRSV Samples

The lung samples were collected from pigs suspected of PRRSV infection in farms located in Hubei Province, followed by homogenization in RPMI-1640 medium (Hyclone, Logan, UT, USA) for RNA extraction and virus isolation. Total RNA extraction from each sample (~1.0 g) was performed using TRIzol reagent (Invitrogen, Waltham, MA, USA), followed by reverse transcription to synthesize cDNA with oligo (dT) primer using Transcriptor First Strand cDNA Synthesis Kit (Roche, Basel, Swiss Confederation). For PCR analysis, a pair of specific primers (Forward: 5′-CTCCTTTGATTGGAATGTTGTG-3′; Reverse: 5′-GATGGCTTGAGCTGAGTATTTTG-3′) were used to amplify a gene fragment in NSP2, which is used for the differential diagnosis of the NADC30-like PRRSV (681 bp), classical PRRSV (1072 bp), and HP-PRRSV (984 bp) strains [27]. The existence of PRRSV RNA was confirmed by using agarose gel electrophoresis to analyze the PCR products.

### 2.2. Amplification and Sequencing of the PRRSV ORF5 Gene

The full-length sequence of the ORF5 gene was amplified using primers (Forward: 5′-TGGCAATTTGAATGTTCAAGTATG-3′; Reverse: 5′-CTGTGCTATCATTGCAGAAGTCGT-3′) [27]. After cloning the amplified ORF5 gene into the pGEM-Teasy vector to obtain the ORF5 plasmid, the plasmid was introduced into *Escherichia coli* DH5α-receptive cells, and positive clones were amplified by PCR. After successful verification, the plasmid was extracted, and then the correct plasmid was sent to Tsingke Biotech (Wuhan, China) for sequencing.

### 2.3. Genetic Evolution Analysis of the PRRSV ORF5 Gene

MAFFT software was applied for a comparative analysis between the sequences of the obtained ORF5 gene and the common reference strains published by GenBank. Mega-X software was used to draw genetic evolutionary trees in accordance with the neighbor-joining algorithm [28], with the background information of reference strains shown in Table 1.

### 2.4. Virus Isolation

To isolate viruses, the above prepared tissue homogenates were frozen/thawed three times, followed by centrifugation (10,000× *g*, 15 min), and subsequent filtration through a 0.22-μm filter. Next, porcine alveolar macrophages (PAMs) were inoculated with the supernatant in a 5% CO_2_ incubator at 37 °C. At 75% cytopathic effects (CPE), PAMs were freeze-thawed together with the culture medium, followed by harvesting the cell lysates and storage at −80 °C for further analysis.

### 2.5. Indirect Immunofluorescence Assay (IFA)

After ~24 h of incubation with the isolated virus, PAMs were fixed with 4% paraformaldehyde for 15 min, followed by 10 min of permeabilization with methanol at room temperature, three PBS washes, and subsequent blocking with 5% BSA. Next, PRRSV N protein expression was detected by using the PRRSV N-specific monoclonal antibody prepared by our lab [26] and Alexa Fluor 488-conjugated donkey anti-mouse IgG (Jackson ImmunoResearch Laboratory, Philadelphia, PA, USA) [29]. Finally, after three PBS washes, the stained cells were visualized with a fluorescent microscope.

### 2.6. Growth Curve Detection

For viral growth curve detection, the infection of PAMs with PRRSV (CHN-HB-2018) was performed in 12-well plates at 0.01 multiplicity of infection (MOI) for 2 h (37 °C). After removing the inoculum and three PBS washes, the plates were supplemented with fresh medium. At 6, 12, 24, 36, and 48 h post infection (hpi), the supernatant and cells were harvested, and a 50% tissue culture infectious dose (TCID_50_) assay was performed to determine viral titers.

### 2.7. Sequencing and Analysis of the PRRSV Genome

The full-length genome of the CHN-HB-2018 strain was amplified using thirteen pairs of primers, followed by cloning the purified PCR products into pEASY-T1 vector. The correct sequence information was obtained for each fragment by using at least three positive bacterial colonies to extract plasmids for subsequent sequencing with the Sanger approach (TsingKe Biotech).

The genome sequence of the CHN-HB-2018 strain was obtained through incorporation of the consistent sequencing results of different fragments using DNAMAN software, and then deposited in the GenBank with accession number MZ043753. MAFFT was used to align the nucleotide and amino acid sequences of the CHN-HB-2018 whole genome and different regions (six structural proteins and 13 nonstructural proteins) with another 42 PRRSV-2 strains (Table 1) [28]. Next, IQ-TREE was used to construct maximum-likelihood (ML) phylogenetic trees [30], and the program automatically determined the best-fitting nucleotide substitution model. Bootstrap values were calculated on 10,000 replicates. The visualization of all trees was performed using Interactive Tree of Life (iTOL) v.4 (http://itol.embl.de/), and Clustal W (Lasergene software, Version 7.1) was used to analyze the nucleotide and amino acid identities of CHN-HB-2018 with the VR-2332, CH-1a, China NADC30 (NADC30_China_), USA NADC30 (NADC30_USA_), 10-10BJ-1, and JXA1 strains, respectively.

### 2.8. Recombinant Analysis

Potential recombinants were investigated by comparing the nucleotide sequence of CHN-HB-2018 with those of reference PRRSV strains (the CH-1a-like strain, HP-PRRSV, and the NADC30-like strain) using RDP V4.24 [31]. Briefly, based on multiple alignments of genomes of reference PRRSV strains, seven methods (RDP, BootScan, GeneConv, SiScan, MaxChi, 3Seq, and Chimera) were applied to evaluate the potential recombinants, with Bonferroni correction and a *p*-value of 0.01 as general settings. 

SimPlot software v.3.5.1 was used for further recombination analysis [32] at a 500 bp window size and a 50 bp step size, with the entire genome of the CHN-HB-2018 strain as the query sequence.

### 2.9. Animal Experiment

Twelve 4-week-old Landrace × Large White × Duroc piglets, each with an average weight of 5.52 kg and an equal distribution of males and females, were used in our animal experiment. Prior to the commencement of the experiment, the sera of piglets were examined for PRRSV antigen using RT-qPCR and PRRSV-specific antibodies through ELISA. Only piglets that tested negative for both PRRSV antigen and PRRSV-specific antibodies were chosen. The piglets in the infected group (n = 7) and control group (n = 5) were inoculated intramuscularly (3 mL) and intranasally (3 mL) with CHN-HB-2018 (1 × 10^6.5^ TCID_50_/mL) and DMEM, respectively.

At 0, 3, 5, 7, 10, 14, and 21 days post-infection (dpi), blood samples were collected for RT-qPCR analysis of viremia and detection of PRRSV-specific antibodies using ELISA kits (Ke Qian Biology, Wuhan, China). Rectal temperature and clinical symptoms (feed intake, respiration, and mental condition) were recorded and the clinical sign scores were quantified daily after infection as described in previous studies [33,34]. The body weight of each piglet was measured at 0, 7, 14, and 21 dpi. At 7 dpi, three of the seven piglets in the infected group were euthanized, and the others were euthanized at 21 dpi. 

At necropsy, lung, lymph node, brain, and tonsil samples were collected for RT-qPCR to detect viral load. Lung samples were fixed in formalin for histopathological analysis and immunohistochemistry (IHC). IHC was performed with a PRRSV N-specific monoclonal antibody.

### 2.10. RT-qPCR

Total RNA was extracted and reverse transcribed into cDNA as described in Section 2.1. qPCR was performed in triplicate in an ABI 7500 real-time PCR system (Applied Biosystems, Foster City, CA, USA) using Power SYBR green PCR master mix. Viral RNA levels were quantified using standard curves.

### 2.11. Statistical Analysis

Statistical analyses were analyzed with an unpaired *t*-test. The significance levels were set at 0.05, 0.01, and 0.001, as indicated by *, **, and ***, respectively. The data were analyzed with GraphPad Prism 8.0 for Windows (GraphPad Software, Inc., La Jolla, CA, USA).

## 3. Results

### 3.1. Epidemiological Investigation of PRRSV in Hubei Province in China during 2017–2018

**Figure 1:**
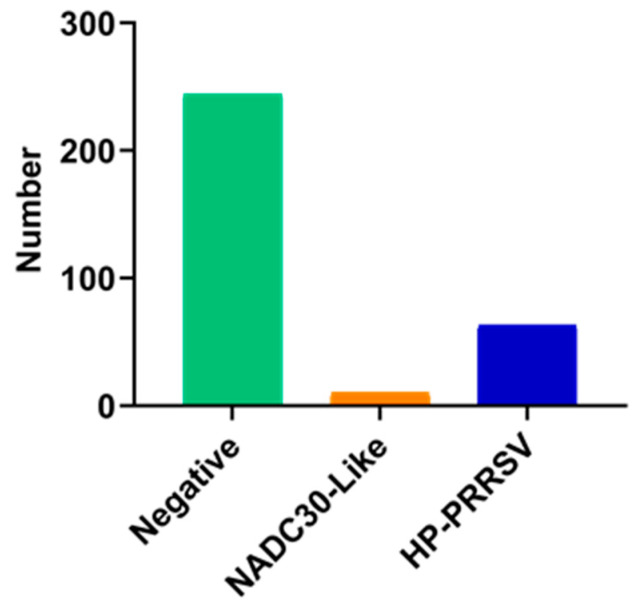
Epidemiological investigation of PRRSV in Hubei Province, China.

From 2017 to 2018, 317 lung tissue samples of respiratory diseased pigs were collected from Hubei Province in China and detected by RT-PCR. The results showed that the PRRSV positive detection rate was 23.03%. Among them, HP-PRRSV accounted for 86.3%, and NADC30-like PRRSV accounted for the remaining 13.7% (Figure 1), indicating that NADC30-like PRRSV was the second most prevalent strain in Hubei Province during 2017–2018, following HP-PRRSV.

### 3.2. Isolation of PRRSV Strain CHN-HB-2018

**Figure 2:**
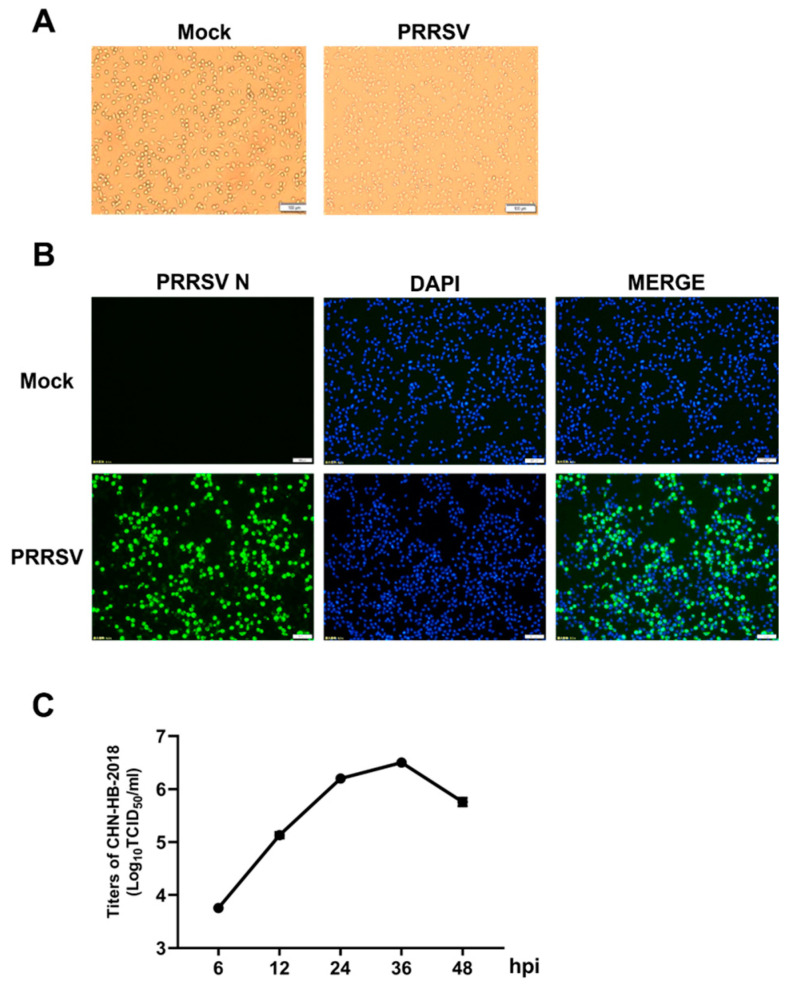
Isolation and identification of PRRSV in PAMs. (**A**) Left: PAMs treated with RPMI 1640 medium. Right: PAMs treated with the supernatant of lung tissue homogenates from a sick pig for 24 h. The images were obtained through phase contrast microscopy. High-power field = 10×. Scale bar = 100 µm. (**B**) IFA detection of PAMs infected with PRRSV CHN-HB-2018 strain. Green: infected cells. Blue: DAPI-stained cell nuclei. (**C**) The one-step growth curve of PRRSV in PAMs.

PAMs were inoculated with the supernatant of lung tissue homogenates obtained from NADC30-like PRRSV-positive pigs. After ~24 h of inoculation, typical PRRSV CPEs were observed (Figure 2A). IFA was performed using a PRRSV N-specific monoclonal antibody to further confirm the presence of PRRSV. As shown in Figure 2B, N protein (green fluorescence signal) was detected in virus-infected cells. Additionally, PRRSV isolate proliferation was confirmed by a growth curve generated via TCID_50_ assays, and viral titer was seen to gradually increase during 6–36 hpi, followed by a slight decline (Figure 2C). Taken together, we successfully isolated a PRRSV strain, hereafter named CHN-HB-2018 with accession number MZ043753 in GenBank.

### 3.3. Whole-Genome Characterization and Phylogenetic Analysis of CHN-HB-2018

**Figure 3:**
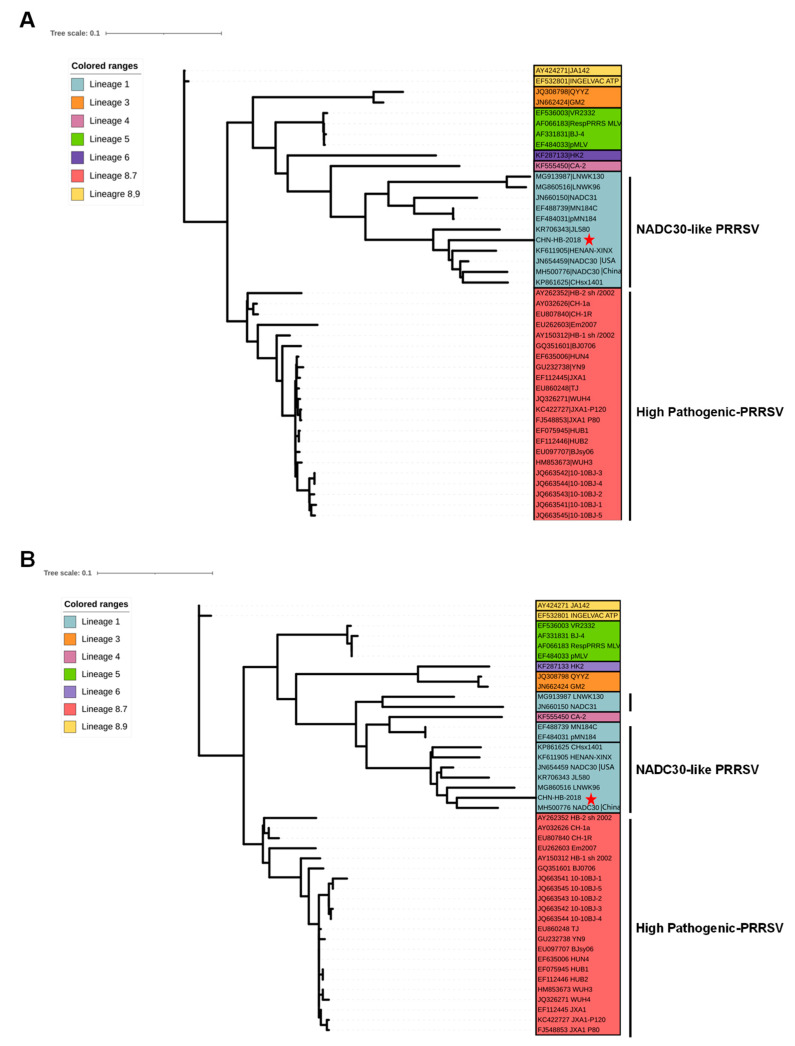
Phylogenetic trees based on (**A**) complete genomes and (**B**) ORF5 nucleotide sequences of PRRSV. The trees consist of seven subgroups, including Lineage 1 (11 strains), Lineage 3 (2 strains), Lineage 4 (1 strain), Lineage 5 (4 strains), Lineage 6 (1 strain), Sublineage 8.7 (22 strains), and Sublineage 8.9 (2 strains). The CHN-HB-2018 strain belongs to Lineage 1, highlighted by a red star.

Sequencing results showed that the CHN-HB-2018 strain, excluding the poly(A) tail, had a full-length genome of 15,001 nucleotides (nt). We analyzed its genetic characteristics by constructing a phylogenetic tree with the complete genome sequences of CHN-HB-2018 and another 42 PRRSV-2 strains (Table 1). Phylogenetic analysis revealed that (*i*) Lineage 1 contains the CHN-HB-2018, China NADC30, USA NADC30, MN184C, pMAN184, NADC31, LNWK96, LNWK130, JL580, HENAN-XINX, and CHsx1401 strains; (*ii*) Lineage 3 includes the GM2 and QYYZ strains; (*iii*) Lineage 4 and 6 contain the CA-2 and HK2 strains, respectively; (*iv*) Lineage 5 consists of the VR-2332, BJ-4, pMLV, and RespPRRS-MLV strains; (*v*) Sublineage 8.7 contains 22 HP-PRRSV strains, including the WUH3, WUH4 strains (previously isolated by our group), and the representative strains of HUN4, JXA1, and TJ; and (*vi*) Sublineage 8.9 contains the JA142 and INGELVAC ATP strains (Figure 3A). As shown above, the CHN-HB-2018 strain was classified within Lineage 1. Additionally, similar phenomena could be observed in most phylogenetic trees of viral proteins, including trees based on nucleotide sequences of NSP2-3, NSP7-8, NSP10-12, ORF2a, and ORF3-7 (Supplementary Figure S1), as well as trees based on the amino acid sequences of NSP1α, NSP2-3, NSP7-8, NSP10-12, GP2a, GP3-5, M, and N proteins (Figure 3B and Supplementary Figure S2), implying that the CHN-HB-2018 strain was a NADC30-like PRRSV strain. In contrast, based on the other phylogenetic trees of viral proteins, including trees from nucleotide sequences of NSP1β and NSP4-5 (Supplementary Figure S1) and trees from amino acid sequences of NSP1β and NSP4-5 (Supplementary Figure S2), the CHN-HB-2018 strain was classified within sublineage 8.7 (JXA1-like/CH-1a-like), indicating its closer relationship with HP-PRRSV at these regions.

Moreover, we investigated the nucleotide identity between CHN-HB-2018 and the other six reference PRRSV strains, including the VR-2332 (a representative PRRSV-2 strain), CH-1a (the first isolated PRRSV strain in China), 10-10BJ-1 (a newly emerged HP-PRRSV strain), JXA1 (a representative HP-PRRSV strain), China NADC30, and USA NADC30 strains. As shown in Table 2, CHN-HB-2018 had the highest complete genome nucleotide identity with NADC30, including USA NADC30 (NADC30_USA_; 92.8% identity) and China NADC30 (NADC30_China_; 90.9% identity), followed by 82.9%, 82.7%, 82.4%, and 81.6% identity with JXA1, CH-1a, 10-10BJ-1, and VR-2332, respectively, further suggesting that CHN-HB-2018 is a novel NADC30-like PRRSV isolate.

The genomic variation of the CHN-HB-2018 strain was further characterized by its comparison with the above six reference PRRSV strains in nucleotide sequences of different coding regions (six structural proteins and 13 nonstructural proteins) and noncoding regions (5′-UTR and 3′-UTR). In Table 2, homology analysis revealed that the CHN-HB-2018 strain had the highest nucleotide identity (92.4–100%) with the USA NADC30 (NADC30_USA_) strain in the regions encoding eight nonstructural proteins (NSP1α, NSP2-3, NSP7-8 and NSP10-12) and all structural proteins (ORF2a and ORF3-7), as well as one noncoding region (3′-UTR). By contrast, in 5′-UTR, NSP1β, NSP4-6, and NSP9, the CHN-HB-2018 strain had the highest nucleotide identity (88.6–97.9%) with the JXA1 strain. These results suggest the potential existence of NADC30-like and HP-PRRSV strain recombination events. However, we noted that high nucleotide identities were also observed across all analyzed PRRSV strains in 5′-UTR, NSP1β, and NSP6. Additionally, the nucleotide identity between the CHN-HB-2018 strain and the USA NADC30 lineage was comparable to that between the CHN-HB-2018 strain and the JXA1 lineage in NSP9. Therefore, while nucleotide identity analyses provide valuable insights, they should only serve as supplements to identifying recombination events. Further recombinant analyses are still necessary.

### 3.4. Amino Acid Variations in NSP2, GP3 and GP5 of CHN-HB-2018

**Figure 4:**
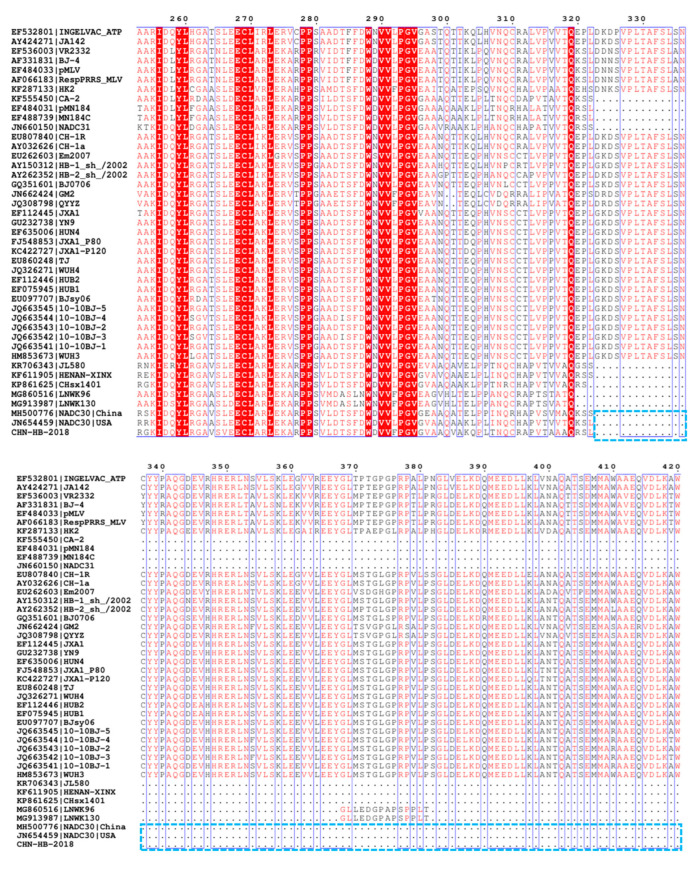

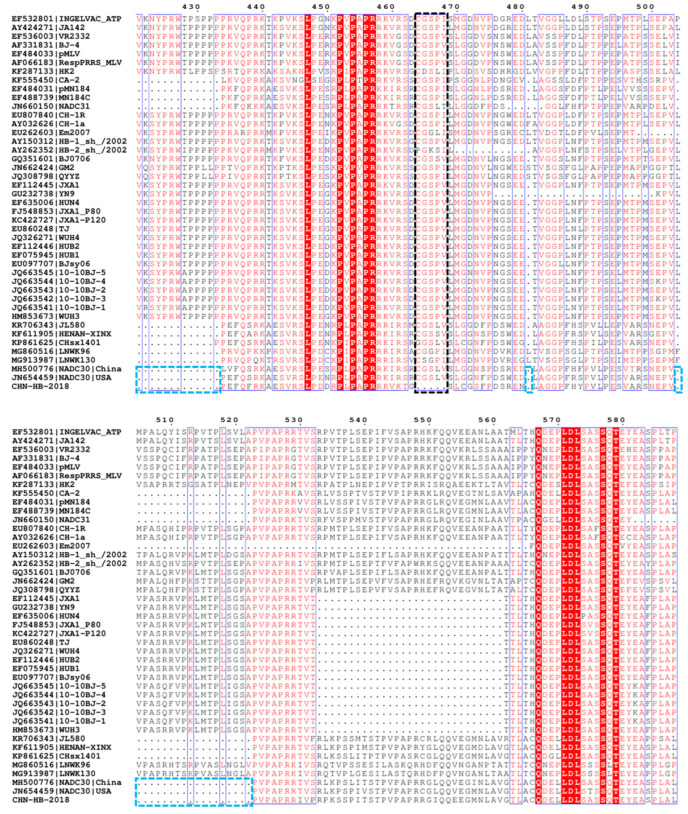
The regions with amino acid deletion in the NSP2 of the CHN-HB-2018 strain versus 42 representative PRRSV strains. The blue boxes indicate a discontinuous deletion of 131-aa (positions 323–433, 481, and 504–522) in the CHN-HB-2018 strain, which is analogous to previous NADC30-like strains. The black box indicates novel a deletion in the CHN-HB-2018 strain at positions 464–468.

NSP2 (the largest nonstructural protein) and GP5 (a major envelope protein) are the most variable PRRSV proteins, so the two viral proteins are commonly used to analyze PRRSV variation and strain classification [17,26]. GP3, a highly soluble glycosylated protein with low homology among PRRSV strains, contains multiple epitope regions [35,36]. In this study, we compared alterations between CHN-HB-2018 and another 42 PRRSV-2 representative strains in amino acid sequences of NSP2, GP3, and GP5. As shown in Figure 4, the CHN-HB-2018 strain showed a discontinuous 131-aa deletion in NSP2 at positions 323–433, 481, and 504–522, which are typical deletions of the NADC30-like strain prevalent in China [24]. Importantly, an additional 5-aa deletion at position 464–468 was observed in the NSP2 of the CHN-HB-2018 strain, which has not been reported before. 

**Figure 5:**
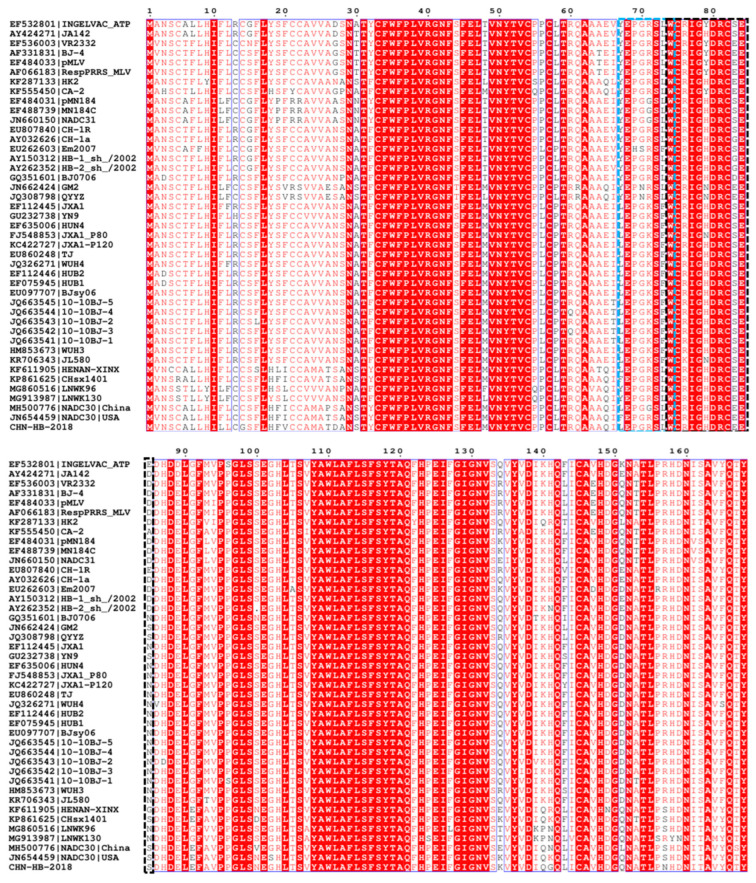
Alignment of amino acid sequences derived from GP3 between the CHN-HB-2018 strain and 42 representative PRRSV strains. Two antigenic epitopes (67 YEPGRSLW 74 and 74 WCRIGHDRCGED 85) are highlighted with blue and black boxes, respectively.

As for GP3, the two key antigenic epitopes (67 YEPGRSLW 74 and 74 WCRIGHDRCGED 85) have previously been reported. In these two epitopes, the CHN-HB-2018 strain exhibited several mutations when compared to the VR2332 strain, but only one amino acid difference at positions 71 and 67 relative to the China NADC30 and USA NADC30 strain, respectively (Figure 5). 

**Figure 6:**
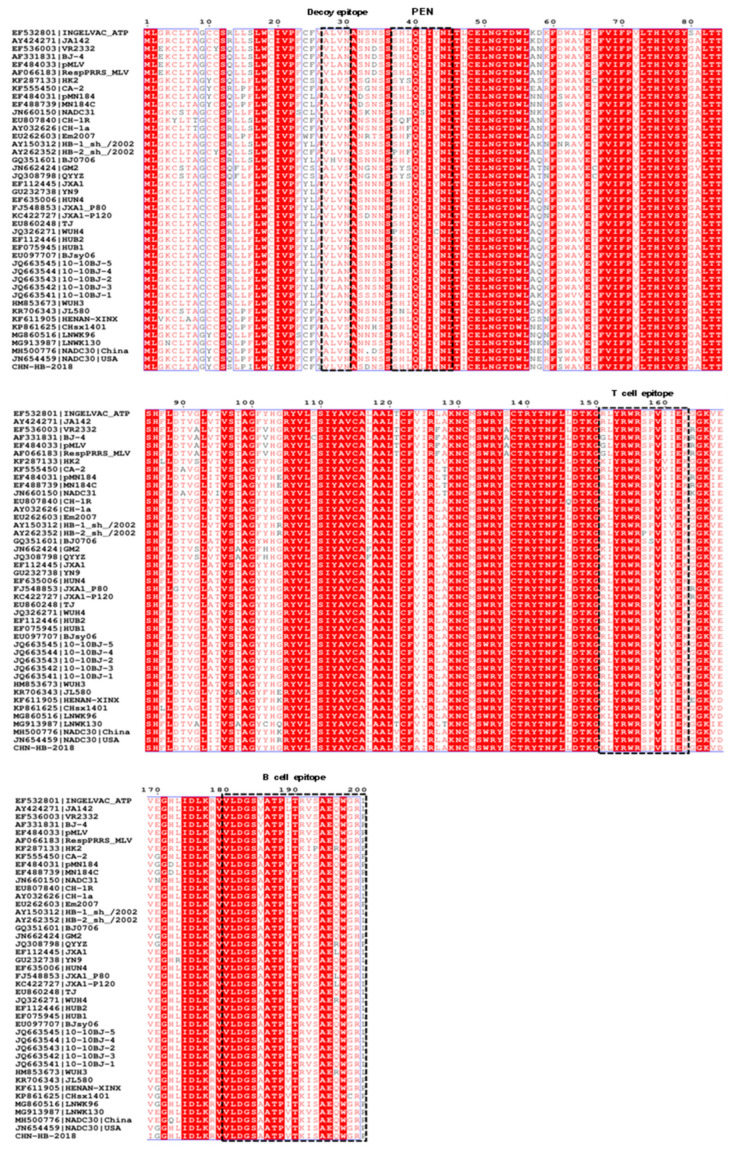
Comparison of GP5 amino acid sequences between the CHN-HB-2018 strain and 42 representative PRRSV strains in the decoy epitope (27 V/ALVN 30), primary neutralizing epitope (PNE) (37 SHF/LQLIYNL 45), T cell epitope (151 RLYRWRSPVIIEK 163), and B cell epitope (180 VLDGSVATPITRVSAEQWGRP 200). These regions are highlighted by four black boxes.

There are several vital epitopes in GP5, such as the B cell epitope (180 VLDGSVATPITRVSAEQWGRP 200), T cell epitope (151 RLYRWRSPVIIEK 163), primary neutralizing epitope (PNE) (37 SHF/LQLIYNL 45), and decoy epitope (27 V/ALVN 30) [37]. As shown in Figure 6, the CHN-HB-2018 strain had two amino acid mutations (S30N and A27V) in the decoy epitope region when compared to the China NADC30 strain, but only one amino acid mutation (A27V) relative to the USA NADC30 strain. Furthermore, the CHN-HB-2018 strain exhibited two amino acid variations (Q13S and R151K), the virulence-related residues in GP5 [38], when compared to the China NADC30 strain, but only one variation (Q13S) relative to the USA NADC30 strain. Additionally, N-linked glycosylation sites (N34 and N51) in the GP5 ectodomain are involved in the production of progeny viruses and neutralizing antibodies [39], where N51 is a conserved glycosylation site, while D34N substitution was observed in the CHN-HB-2018 strain versus the China NADC30 strain. Furthermore, when compared to both the USA NADC30 and China NADC30 strains, the CHN-HB-2018 strain showed an additional amino acid mutation (N32S) at a potential glycosylation site, and another two specific point mutations at positions F16I and C19Y.

### 3.5. Gene Recombination Analysis

**Figure 7:**
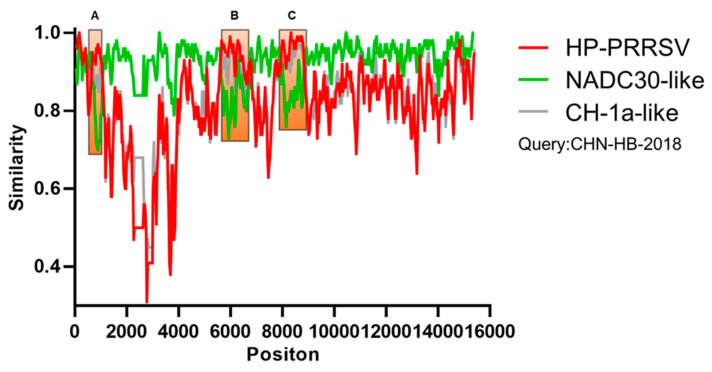
Genome recombination analysis of the CHN-HB-2018 strain. The Y-axis indicates the percentage similarity of the query sequence (CHN-HB-2018) to the HP-PRRSV (red), NADC30-like (green), and CH-1a-like (gray) strains.

In gene recombination analysis, we used the entire genomes of the CHN-HB-2018 strain and reference strains (the HP-PRRSV, NADC30-like, and CH-1a-like strains) as query and reference sequences, respectively. The CHN-HB-2018 strain was shown to be closely related overall to the NADC30-like strain, but more closely related to HP-PRRSV at nt 600–1000 (NSP1β), nt 5601–6651 (NSP4-6), and nt 7851–8801 (NSP9) (Figure 7). Therefore, the CHN-HB-2018 strain can be assumed to originate from NADC30-like and HP-PRRSV recombination events, partially agreeing with the conclusions of phylogenetic analysis and homology analysis.

### 3.6. Pathogenicity Analysis

**Figure 8:**
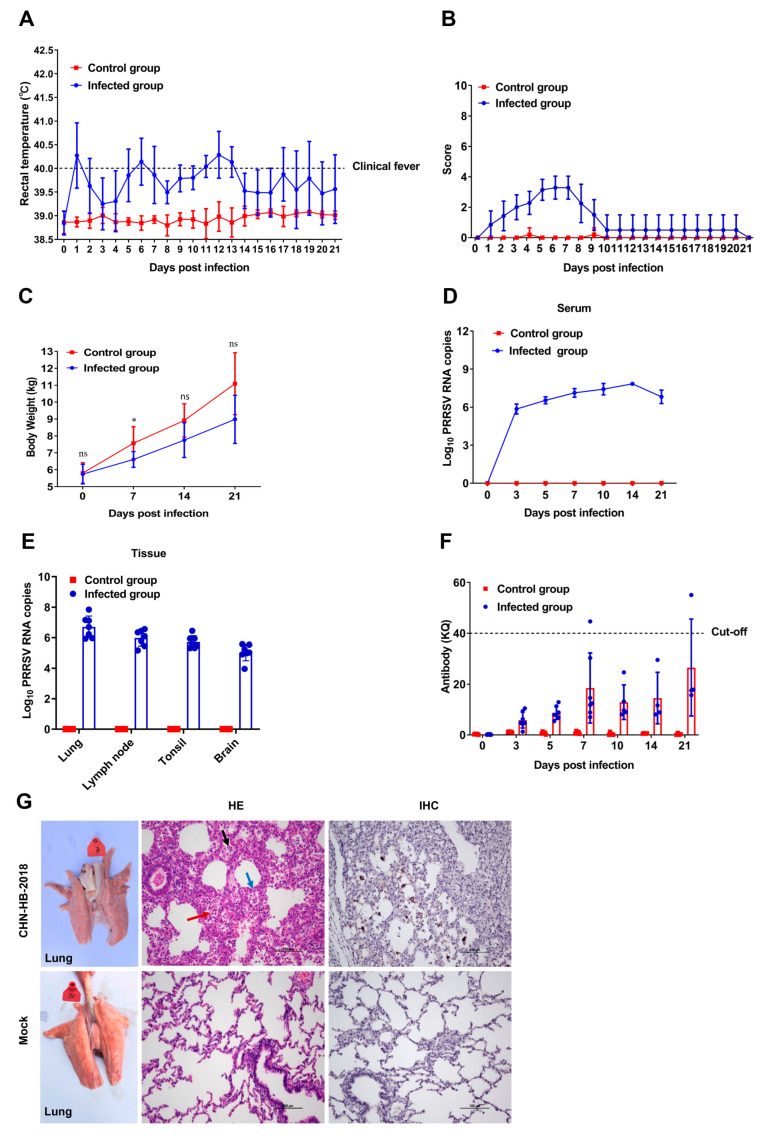
Pathogenicity analysis of the CHN-HB-2018 strain in piglets. Rectal temperature (**A**), clinical sign scores (**B**), body weight (**C**), serum viral RNA copy number (**D**), tissue viral RNA copy number (**E**), PRRSV-specific antibody levels (**F**), and histopathological/immunohistochemical analysis (**G**). Values in (**A**–**F**) are shown as mean ± SD. Black arrow: neutrophilic infiltration; Red arrow: alveolar macrophage infiltration; Blue arrow: alveolar epithelial cell hyperplasia. * *p* < 0.05; ns: no significance.

The pathogenicity results showed that the piglets inoculated with PRRSV strain CHN-HB-2018 (infected group) displayed mild clinical signs, and no typical clinical symptoms were observed in piglets inoculated with DMEM (control group). Specifically, in the infected group, rectal temperature reached 40.3 °C at 1 dpi, causing fever (over 40 °C), and hovered over 40 °C from 5 to 13 dpi, followed by a drop and return to normal (below 40 °C), while in the control group, rectal temperature stayed normal and lower than that of the infected group throughout the experiment (Figure 8A). For scores of other clinical signs, the infected piglets manifested decreased appetite, breathlessness, and cough from 1 to 9 dpi, and returned to normal from 10 dpi, but the control piglets showed no obvious abnormality during the 21-day experiment (Figure 8B). In terms of body weight (Figure 8C), less body weight was gained for infected piglets versus control piglets.

Viremia was detected in infected piglets at 3 dpi and reached a maximum at 14 dpi, followed by a slight drop (Figure 8D). Additionally, viral load was also detected in the lung, lymph node, brain, and tonsil tissues of all infected piglets, but not in control piglets (Figure 8E). PRRSV-specific antibodies in serum were detected using commercially available ELISA kits. As shown in Figure 8F, despite a gradual increase in the levels of antibodies, only two antibody-positive (KQ > 40) piglets were found after 7 dpi. 

All the infected and control piglets survived the entire experimental period and were euthanized at the indicated time points. At necropsy, the infected piglets exhibited local hemorrhages, widened intermass, and flesh changed in the lungs, but no such observations occurred in control piglets (Figure 8G). Histopathologically, the CHN-HB-2018 strain caused alveolar wall thickening, alveolar epithelial cell hyperplasia, and alveolar neutrophil and macrophage infiltration, in contrast to no obvious pathological changes observed in the lungs of control piglets. Additionally, IHC staining detected PRRSV antigens in the lungs of infected piglets, but not in control lungs (Figure 8G).

## 4. Discussion

Since first emerging in 1995, PRRS has been one of the most common pig diseases in China. Due to its genetic diversity in the field and the insufficient effectiveness of commercially available vaccines against newly emerging PRRSV strains, there is an acute need for regular surveillance to obtain valuable information for effective PRRS disease control. In this study, HP-PRRSV and NADC30-like strains were observed to account for 86.3% and 13.7% of PRRSV cases during 2017–2018 in Hubei Province, China, enriching the evidence of the transition of the predominant circulating PRRSV strain from HP-PRRSV strains to the NADC30-like strain. Furthermore, the month of May 2018 witnessed the eruption of a severe respiratory disease (a mortality rate of ~20%) in a pig farm in Hubei, China, with the main clinical symptoms including anorexia, fever, cough, cyanosis, etc., and no improvement observed after treatment with florfenicol, aureomycin, oxytetracycline, and other drugs. From this pig farm, we isolated the NADC30-like PRRSV strain of CHN-HB-2018 and explored its molecular evolution characteristics by comparing its complete nucleotide sequence to those of 42 representative PRRSV strains isolated from China and other countries. The results showed that the CHN-HB-2018 strain was in the same evolutionary tree branch with the NADC30 strain, belonging to Lineage 1 of PRRSV-2.

In the PRRSV genome, the NSP2 gene has been shown to have the highest genetic diversity [40], and its protease activity plays a crucial role in viral replication and host immunity modulation [41]. A comparison with VR-2332 showed three discontinuous deletions (131 aa) in the NSP2 of the CHN-HB-2018 strain, similar to the case in the NADC30 strain, which is usually used to distinguish them from other PRRSV strains as a molecular marker, such as HP-PRRSV with 30-aa deletion in NSP2 [23]. Importantly, unlike other NADC30 or NADC30-like strains, the NSP2 of the CHN-HB-2018 strain was found to have an additional 5-aa deletion from position 464 to 468, which has not previously been reported. In fact, we have isolated seven NADC30-like PRRSV strains in PAMs, but failed to find this 5-aa deletion in the other six PRRSV strains. Interestingly, these six PRRSV strains could not be passaged more than twice in Marc-145 cells, while the CHN-HB-2018 strain could not only infect PAMs, but also be stably passaged in Marc-145 cells, inferring that this novel amino acid deficiency at 464–468 aa sites in NSP2 may contribute to the difference in the cell tropism of various PRRSV strains. However, this hypothesis needs to be verified through further experiments. 

ORF5, which encodes the GP5 protein, is also a frequently mutated gene of PRRSV, leading to its common use in analysis of PRRSV genetic variations. The decoy epitope (27 V/ALVN 30) and PNE (37 SHF/LQLIYNL 45) of GP5 are key to immune responsiveness induction during PRRSV infection [42]. In our study, compared to the China NADC30 strain (accession number MH500776), the CHN-HB-2018 strain showed two amino acid substitutions (S30N and A27V) in the decoy epitope. Furthermore, two mutants at virulence-related residues (Q13S and R151K) existed in GP5 of the CHN-HB-2018 strain. N-glycosylation is not only involved in immune escape and neutralizing antibody production, but also in the manipulation of viral adsorption, invasion, and budding processes. When compared to the China NADC30 strain (N32, N44, and N51), the CHN-HB-2018 strain (N30, N34, N44, and N51) showed an increase in N-glycosylation site number, which may affect PRRSV virulence. Notably, when compared to the USA NADC30 strain (accession number JN654459), the CHN-HB-2018 strain showed fewer amino acid mutations in GP5, with only one mutation (A27V) in decoy epitope and one mutation (Q13S) at virulence-related residue, and more similar N-glycosylation sites (N30, N32, N34, N44, and N51 in the USA NADC30 strain) in comparison to the China NADC30 strain, indicating that in terms of GP5, the CHN-HB-2018 strain was more closely related to the USA NADC30 strain than the China NADC30 strain. Importantly, homology analysis also revealed that CHN-HB-2018 shared higher identity with the USA NADC30 strain (92.8%) than with the China NADC30 strain (90.9%). Therefore, one may speculate that the CHN-HB-2018 strain was a novel NADC30-like strain prior to the emergence (2013) of the China NADC30 strain (accession number: MH500776) in 2013, which requires confirmation by means of more systematic geographical and temporal analysis. 

Recombination between various sublineages/lineages of PRRSV is a vital mechanism for its new strain creation. Since the emergence of NADC30-like PRRSV in China in 2013, many recombination events involving PRRSV-2 strains have occurred in the field, leading to frequent reports of genetic exchanges of the NADC30 strain with previously major circulating PRRSV strains in China [24]. In this study, a novel recombinant PRRSV strain was isolated, which might be derived from NADC30-like strains and HP-PRRSV recombination events, with a recombination region located in HP-PRRSV NSP1β (nt 600–1000), NSP4-6 (nt 5601–6651), and NSP9 (nt 7851–8801). In China, recombination hotspots have been reported to mainly occur in NSP1-2 (nt 1–2300), NSP8 (nt 7600–7700), NSP9 (nt 7900–8000), and GP2 (nt 12,100–12,200), respectively [43]. In this study, a new recombination site was found at nt 5601–6651, suggesting that the PRRSV recombination patterns in China may be becoming increasingly complex. Additionally, NSP1, NSP4, and NSP9 are three important nonstructural proteins related to PRRSV replication, suggesting that the recombination events among these genes might alter the replication efficiency of the CHN-HB-2018 strain. 

The pathogenicity of recombinant NADC30-like PRRSV has been found to be intermediate between the two parental strains. In the present study, we demonstrated that CHN-HB-2018 was a recombinant strain with NADC30 and HP-PRRSV as parental strains. It is well known that HP-PRRSV infection typically causes high fever (41~42 °C), high morbidity, and high mortality, as well as interstitial pneumonia and viremia [34]. However, although NADC30 PRRSV can result in interstitial pneumonia and viremia, it commonly only triggers mild fever and does not lead to mortality. Our animal experiments showed that CHN-HB-2018 did not cause piglet mortality, but did lead to viremia, interstitial pneumonia, and mild fever (around 40 °C). These observed symptoms closely resembled those induced by the NADC30 strain [18]. Therefore, when compared to HP-PRRSV, the CHN-HB-2018 strain is mildly pathogenic. However, despite moderate pathogenicity for most NADC30-like strains, some NADC30-like PRRSV strains vary in their pathogenicity. For example, JL580, another NADC30 and HP-PRRSV recombinant strain, can cause the death of all piglets within 14 days post-inoculation, indicating that JL580, unlike CHN-HB-2018, is a highly pathogenic strain closely related to HP-PRRSV [17]. However, HNyc15 and Chsx1401, two PRRSV strains recombined from the NADC30-like strain and classical strains, are moderately pathogenic [44,45,46,47].

At present, there is no vaccine specifically designed for NADC30-like PRRSV, and the currently available PRRSV vaccines offer only limited cross-protection. The CHN-HB-2018 strain could be stably transmitted in Marc-145 cells, and pathogenicity analysis showed that it is moderately virulent, making it a potential candidate for the development of a vaccine against NADC30-like PRRSV.

Herein, we demonstrated the prevalence of the NADC30-like PRRSV strain in Hubei Province during 2017–2018 by means of RT-PCR detection and the analysis of 317 lung tissue samples from respiratory diseased pigs. Additionally, a novel PRRSV strain, designated as CHN-HB-2018, was isolated. Phylogenetic, genetic variation, and recombination analyses revealed that the CHN-HB-2018 strain was a NADC30-like strain derived from possible NADC30 and HP-PRRSV recombination. Notably, CHN-HB-2018 had a 5-aa deletion in NSP2, which has not previously been reported. Animal experiments showed that CHN-HB-2018 is mildly pathogenic. This work facilitates an understanding of the NADC30-like PRRSV evolutionary mechanism in China.

## 5. Conclusions

In summary, we analyzed the epidemiology of PRRSV and found that the NADC30-like strain was the second major prevalent PRRSV strain after HP-PRRSV in Hubei Province during 2017–2018. We further isolated CHN-HB-2018, a NADC30-like PRRSV strain from a farm in Hubei Province. Amino acid variation analysis found a previously unreported 5-aa deletion in its NSP2. Additionally, recombination analysis revealed CHN-HB-2018 to be a potential NADC30-like and HP-PRRSV recombinant strain. Pathogenic experimental results showed that the pathogenicity of the CHN-HB-2018 strain was mild in piglets. Our results not only facilitate an understanding of NADC30-like PRRSV evolutionary characteristics in China, but also provide useful information for PRRS prevention or control.

## Figures and Tables

**Table 1:** The 42 representative PRRSV strains used for sequence comparative analysis.

NO.	GenBank No.	Strains	Reported Date	Country	Genealogy
1	AF331831.1	BJ-4	2016/7/14	China	Lineage1
2	KR706343	JL580	2013/01/01	China	Lineage1
3	KF611905	HENAN-XINX	2013/01/01	China	Lineage1
4	KP861625	CHsx1401	2014/08/30	China	Lineage1
5	EF484031	MN184C	2008/02/26	USA	Lineage1
6	MH500776	NADC30	2017/10/01	China	Lineage1
7	JN654459	NADC30	2011/09/07	USA	Lineage1
8	JN660150	NADC31	2008/01/01	USA	Lineage1
9	MG860516	LNWK96	2017/01/01	China	Lineage1
10	MG913987	LNWK130	2017/01/01	China	Lineage1
11	KF484031	PMN184	2008/2/26	USA	Lineage1
12	JQ308798	QYYZ	2011/01/16	China	Lineage3
13	JN662424	GM2	2011/01/01	China	Lineage3
14	KF555450	CA-2	2014/4/30	South Korea	Lineage4
15	EF484033	RespPRRS-MLV	2008/02/26	USA	Lineage5
16	U87392.3	VR-2332	1997/01/28	USA	Lineage5
17	EF484033	pMLV	2008/2/26	USA	Lineage5
18	KF287133	HK2	2014/4/10	Hong Kong	Lineage6
19	GQ351601	BJ0706	2007/06/01	China	Lineage8
20	EU860248	TJ	2006/10/01	China	Lineage8
21	FJ548853	JXA1-P80	2016/07/24	China	Lineage8
22	KC422727	JXA1-P120	2009/01/01	China	Lineage8
23	EF635006	HUN4	2016/07/23	China	Lineage8
24	AY032626	CH-1a	2016/07/22	China	Lineage8
25	EU262603	Em2007	2016/07/26	China	Lineage8
26	AY424271	JA142	2016/07/26	USA	Lineage8
27	EF112445	JXA1	2016/07/14	China	Lineage8
28	HM853673	WUH3	2008/09/01	China	Lineage8
29	JQ326271	WUH4	2011/09/01	China	Lineage8
30	JQ663545	10-10BJ-5	2010/01/01	China	Lineage8
31	JQ663544	10-10BJ-4	2010/01/01	China	Lineage8
32	JQ663543	10-10BJ-2	2010/01/01	China	Lineage8
33	JQ663542	10-10BJ-3	2010/01/01	China	Lineage8
34	JQ663541	10-10BJ-1	2010/01/01	China	Lineage8
35	AY150312	HB-1(sh)/2002	2002/07/24	China	Lineage8
36	AY262352	HB-2(sh)/2002	2002/11/10	China	Lineage8
37	EF532801	INGELVAC ATP	2014/5/30	USA	Lineage8
38	EU807840	CH-1R	2016/7/26	China	Lineage8
39	GU232738	YN9	2008/11/14	China	Lineage8
40	EF075945	HUB1	2016/7/14	China	Lineage8
41	EF112446	HUB2	2007/7/18	China	Lineage8
42	EU097707	BJsy06	2016/7/26	China	Lineage8

**Table 2:** Homology analysis of the CHN-HB-2018 and reference PRRSV strains.

Region	VR-2332	CH-1a	JXA1	10-10BJ-1	NADC30_China_	NADC30_USA_
Complete	81.6	82.7	82.9	82.4	90.9	92.8
5′UTR	90.1	94.8	95.3	94.2	89.5	91.1
NSP1α	90.6	92.2	91.2	90.8	90.6	92.4
NSP1β	84.2	85.7	88.6	87.3	83.7	86.0
NSP2	65.8	63.6	63.9	63.3	90.5	93.2
NSP3	84.7	84.2	83.1	82.6	91.4	93.8
NSP4	87.7	92.2	95.4	95.1	82.0	83.2
NSP5	86.3	90.2	92.5	91.4	84.7	87.3
NSP6	97.9	95.8	97.9	97.9	91.7	95.8
NSP7	84.2	81.3	80.7	81.3	92.3	93.8
NSP8	88.4	87.0	86.2	87.7	92.8	95.7
NSP9	88.6	89.8	90.9	90.2	89.6	90.8
NSP10	85.7	85.9	85.3	85.2	93.7	95.6
NSP11	88.8	90.4	88.6	87.6	93.4	94.2
NSP12	87.4	87.6	87.1	86.1	93.0	95.6
ORF2a	88.5	86.8	86.1	86.1	93.3	94.4
ORF3	82.5	82.7	83.1	82.2	92.7	95.3
ORF4	87.2	87.7	87.2	87.4	94.6	96.8
ORF5	84.1	84.6	84.2	83.1	91.5	92.4
ORF6	89.9	88.2	89.5	89.3	97.0	97.3
ORF7	91.9	89.9	89.2	90.1	94.4	94.9
3′UTR	92.1	79.8	75.8	75.8	97.8	100

## Data Availability

The data underlying this article are available in this manuscript and in its online Supporting Information.

## Supplementary Materials

The following supporting information can be downloaded at: https://www.mdpi.com/article/10.3390/v16060993/s1, Figure S1: Phylogenetic trees based on the nucleotide sequences of viral proteins of PRRSV; Figure S2: Phylogenetic trees based on the amino acid sequences of viral proteins of PRRSV.
