# Supplementary material for: Genetic Characterization and Pathogenicity of a Recombinant Porcine Reproductive and Respiratory Syndrome Virus Strain in China
Source: Viruses. 2024 Jun 20;16(6):993. doi: 10.3390/v16060993 (PMC11209116; doi:10.3390/v16060993)
Supplement: Supplementary file 1 [file viruses-16-00993-s001.zip › Supplementary Figures.pdf]

Supplementary material

Supplementary Figure S1. Phylogenetic trees based on the nucleotide sequences of viral proteins of PRRSV

Supplementary Figure S2. Phylogenetic trees based on the amino acid sequences of viral proteins of PRRSV

Supplementary Figure S1

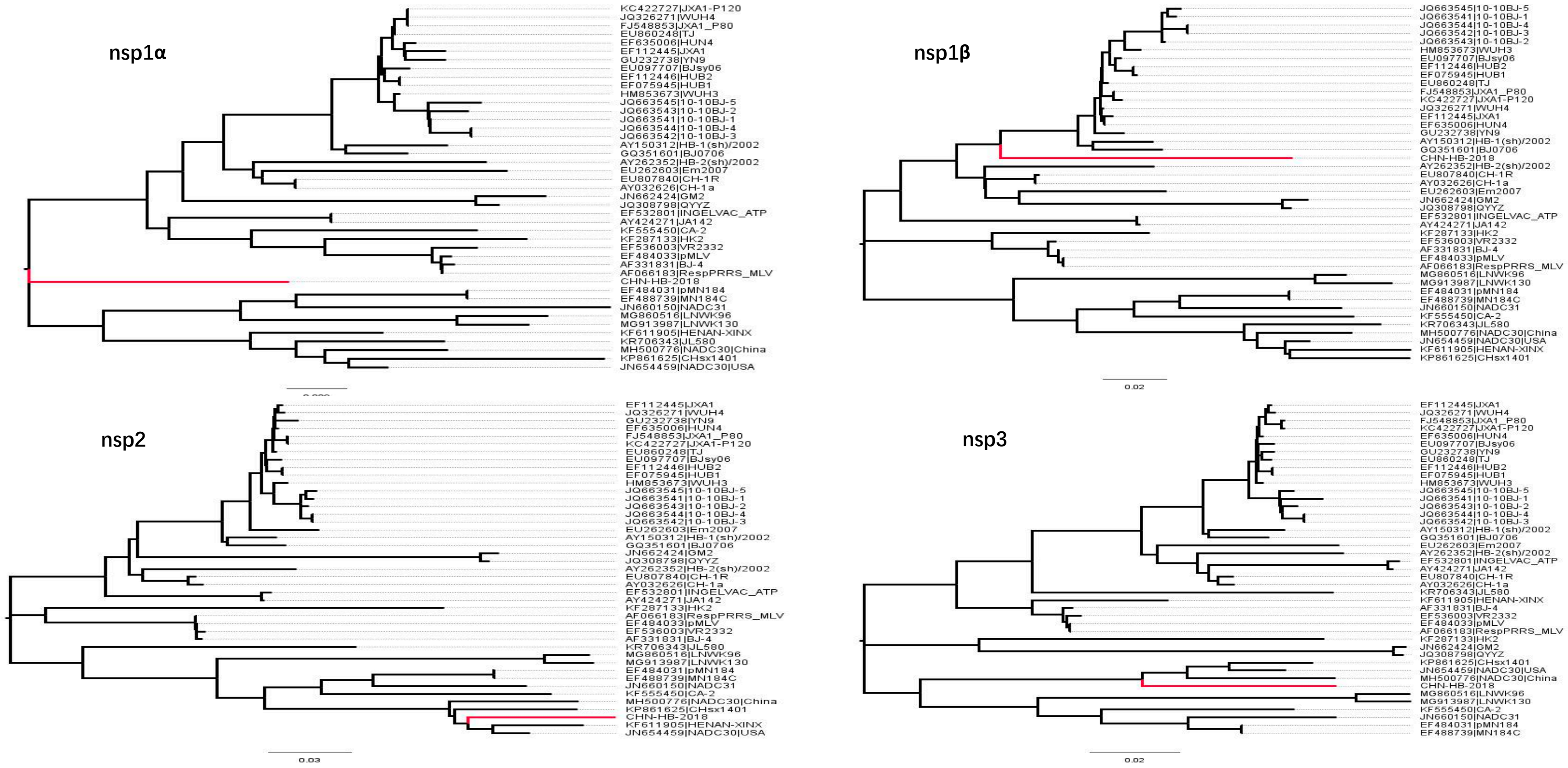

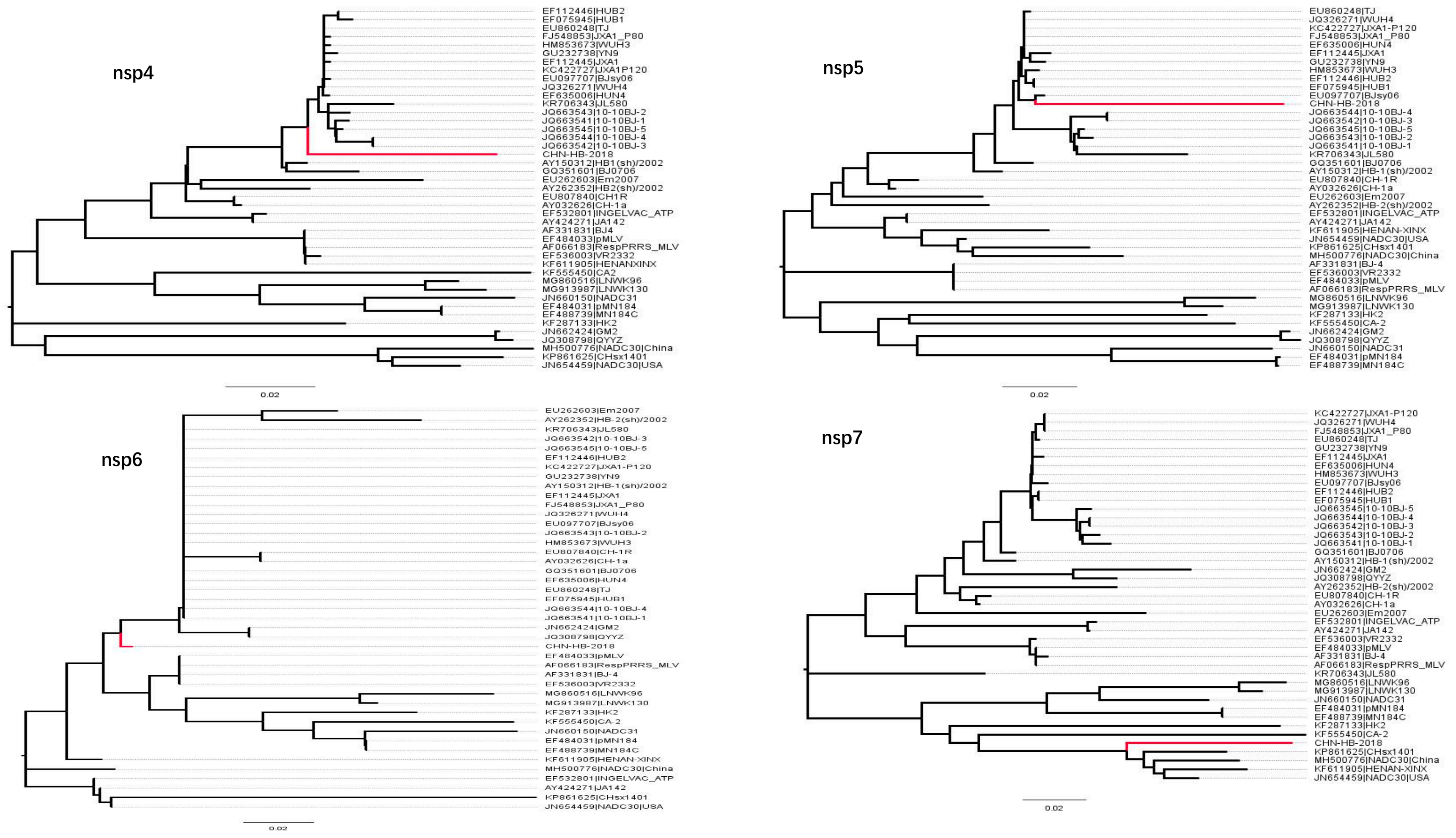
nsp4
EF112446|HUB2
EF075945|HUB1
EU860248|TJ
FJ548853|JXA1_P80
HM853673|WUH3
GU232738|YN9
EF112445|JXA1
KC422727|JXA1P120
EU097707|BJsy06
JQ326271|WUH4
EF635006|HUN4
KR706343|JL580
JQ663543|10-10BJ-2
JQ663541|10-10BJ-1
JQ663545|10-10BJ-5
JQ663544|10-10BJ-4
JQ663542|10-10BJ-3
CHN-HB-2018
AY150312|HB1(sh)/2002
GQ351601|BJ0706
EU262603|Em2007
AY262352|HB2(sh)/2002
EU807840|CH1R
AY032626|CH-1a
EF532801|INGELVAC_ATP
AY424271|JA142
AF331831|BJ4
EF484033|pMLV
AF066183|RespPRRS_MLV
EF536003|VR2332
KF611905|HENANXINX
KF555450|CA2
MG860516|LNWK96
MG913987|LNWK130
JN660150|NADC31
EF484031|pMN184
EF488739|MN184C
KF287133|HK2
JN662424|GM2
JQ308798|QYYZ
MH500776|NADC30|China
KP861625|CHsx1401
JN654459|NADC30|USA
0.02
nsp5
EU860248|TJ
JQ326271|WUH4
KC422727|JXA1-P120
FJ548853|JXA1_P80
EF635006|HUN4
EF112445|JXA1
GU232738|YN9
HM853673|WUH3
EF112446|HUB2
EF075945|HUB1
EU097707|BJsy06
CHN-HB-2018
JQ663544|10-10BJ-4
JQ663542|10-10BJ-3
JQ663545|10-10BJ-5
JQ663543|10-10BJ-2
JQ663541|10-10BJ-1
KR706343|JL580
GQ351601|BJ0706
AY150312|HB-1(sh)/2002
EU807840|CH-1R
AY032626|CH-1a
EU262603|Em2007
AY262352|HB-2(sh)/2002
EF532801|INGELVAC_ATP
AY424271|JA142
KF611905|HENAN-XINX
JN654459|NADC30|USA
KP861625|CHsx1401
MH500776|NADC30|China
AF331831|BJ-4
EF536003|VR2332
EF484033|pMLV
AF066183|RespPRRS_MLV
MG860516|LNWK96
MG913987|LNWK130
KF287133|HK2
KF555450|CA-2
JN662424|GM2
JQ308798|QYYZ
JN660150|NADC31
EF484031|pMN184
EF488739|MN184C
0.02
nsp6
EU262603|Em2007
AY262352|HB-2(sh)/2002
KR706343|JL580
JQ663542|10-10BJ-3
JQ663545|10-10BJ-5
EF112446|HUB2
KC422727|JXA1-P120
GU232738|YN9
AY150312|HB-1(sh)/2002
EF112445|JXA1
FJ548853|JXA1_P80
JQ326271|WUH4
EU097707|BJsy06
JQ663543|10-10BJ-2
HM853673|WUH3
EU807840|CH-1R
AY032626|CH-1a
GQ351601|BJ0706
EF635006|HUN4
EU860248|TJ
EF075945|HUB1
JQ663544|10-10BJ-4
JQ663541|10-10BJ-1
JN662424|GM2
JQ308798|QYYZ
CHN-HB-2018
EF484033|pMLV
AF066183|RespPRRS_MLV
AF331831|BJ-4
EF536003|VR2332
MG860516|LNWK96
MG913987|LNWK130
KF287133|HK2
KF555450|CA-2
JN660150|NADC31
EF484031|pMN184
EF488739|MN184C
KF611905|HENAN-XINX
MH500776|NADC30|China
EF532801|INGELVAC_ATP
AY424271|JA142
KP861625|CHsx1401
JN654459|NADC30|USA
0.02
nsp7
KC422727|JXA1-P120
JQ326271|WUH4
FJ548853|JXA1_P80
EU860248|TJ
GU232738|YN9
EF112445|JXA1
EF635006|HUN4
HM853673|WUH3
EU097707|BJsy06
EF112446|HUB2
EF075945|HUB1
JQ663545|10-10BJ-5
JQ663544|10-10BJ-4
JQ663542|10-10BJ-3
JQ663543|10-10BJ-2
JQ663541|10-10BJ-1
GQ351601|BJ0706
AY150312|HB-1(sh)/2002
JN662424|GM2
JQ308798|QYYZ
AY262352|HB-2(sh)/2002
EU807840|CH-1R
AY032626|CH-1a
EU262603|Em2007
EF532801|INGELVAC_ATP
AY424271|JA142
EF536003|VR2332
EF484033|pMLV
AF331831|BJ-4
AF066183|RespPRRS_MLV
KR706343|JL580
MG860516|LNWK96
MG913987|LNWK130
JN660150|NADC31
EF484031|pMN184
EF488739|MN184C
KF287133|HK2
KF555450|CA-2
CHN-HB-2018
KP861625|CHsx1401
MH500776|NADC30|China
KF611905|HENAN-XINX
JN654459|NADC30|USA
0.02

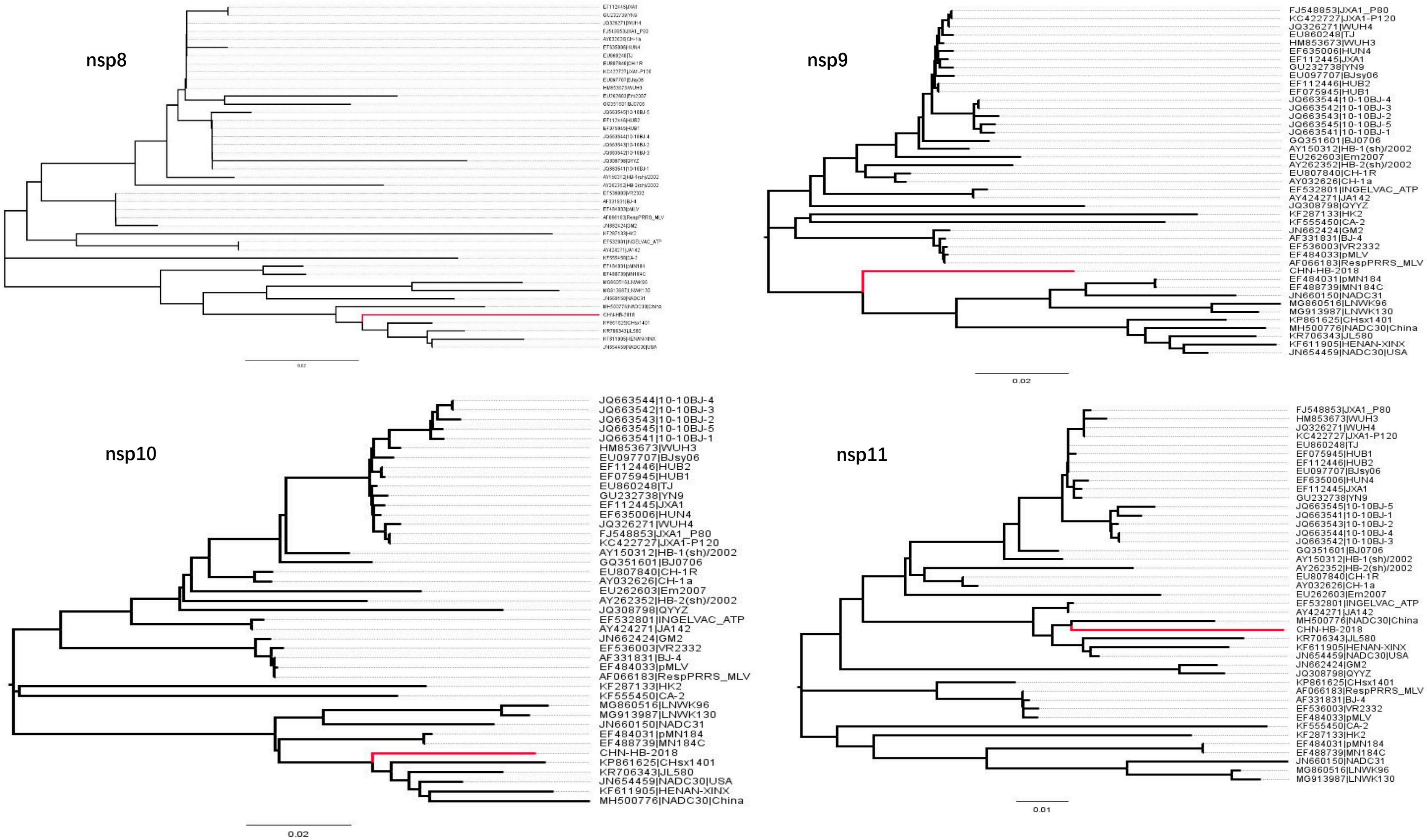

nsp8
nsp9
FJ548853|JXA1_P80
KC422727|JXA1-P120
JQ326271|WUH4
EU860248|TJ
HM853673|WUH3
EF635006|HUN4
EF112445|JXA1
GU232738|YN9
EU097707|BJsy06
EF112446|HUB2
EF075945|HUB1
JQ663544|10-10BJ-4
JQ663542|10-10BJ-3
JQ663543|10-10BJ-2
JQ663545|10-10BJ-5
JQ663541|10-10BJ-1
GQ351601|BJ0706
AY150312|HB-1(sh)/2002
EU262603|Em2007
AY262352|HB-2(sh)/2002
EU807840|CH-1R
AY032626|CH-1a
EF532801|INGELVAC_ATP
AY424271|JA142
JQ308798|QYYZ
KF287133|HK2
KF555450|CA-2
JN662424|GM2
AF331831|BJ-4
EF536003|VR2332
EF484033|pMLV
AF066183|RespPRRS_MLV
CHN-HB-2018
EF484031|pMN184
EF488739|MN184C
JN660150|NADC31
MG860516|LNWK96
MG913987|LNWK130
KP861625|CHsx1401
MH500776|NADC30|China
KR706343|JL580
KF611905|HENAN-XINX
JN654459|NADC30|USA
0.02
nsp10
JQ663544|10-10BJ-4
JQ663542|10-10BJ-3
JQ663543|10-10BJ-2
JQ663545|10-10BJ-5
JQ663541|10-10BJ-1
HM853673|WUH3
EU097707|BJsy06
EF112446|HUB2
EF075945|HUB1
EU860248|TJ
GU232738|YN9
EF112445|JXA1
EF635006|HUN4
JQ326271|WUH4
FJ548853|JXA1_P80
KC422727|JXA1-P120
AY150312|HB-1(sh)/2002
GQ351601|BJ0706
EU807840|CH-1R
AY032626|CH-1a
EU262603|Em2007
AY262352|HB-2(sh)/2002
JQ308798|QYYZ
EF532801|INGELVAC_ATP
AY424271|JA142
JN662424|GM2
EF536003|VR2332
AF331831|BJ-4
EF484033|pMLV
AF066183|RespPRRS_MLV
KF287133|HK2
KF555450|CA-2
MG860516|LNWK96
MG913987|LNWK130
JN660150|NADC31
EF484031|pMN184
EF488739|MN184C
CHN-HB-2018
KP861625|CHsx1401
KR706343|JL580
JN654459|NADC30|USA
KF611905|HENAN-XINX
MH500776|NADC30|China
0.02
nsp11
FJ548853|JXA1_P80
HM853673|WUH3
JQ326271|WUH4
KC422727|JXA1-P120
EU860248|TJ
EF075945|HUB1
EF112446|HUB2
EU097707|BJsy06
EF635006|HUN4
EF112445|JXA1
GU232738|YN9
JQ663545|10-10BJ-5
JQ663541|10-10BJ-1
JQ663543|10-10BJ-2
JQ663544|10-10BJ-4
JQ663542|10-10BJ-3
GQ351601|BJ0706
AY150312|HB-1(sh)/2002
AY262352|HB-2(sh)/2002
EU807840|CH-1R
AY032626|CH-1a
EU262603|Em2007
EF532801|INGELVAC_ATP
AY424271|JA142
MH500776|NADC30|China
CHN-HB-2018
KR706343|JL580
KF611905|HENAN-XINX
JN654459|NADC30|USA
JN662424|GM2
JQ308798|QYYZ
KP861625|CHsx1401
AF066183|RespPRRS_MLV
AF331831|BJ-4
EF536003|VR2332
EF484033|pMLV
KF555450|CA-2
KF287133|HK2
EF484031|pMN184
EF488739|MN184C
JN660150|NADC31
MG860516|LNWK96
MG913987|LNWK130
0.01

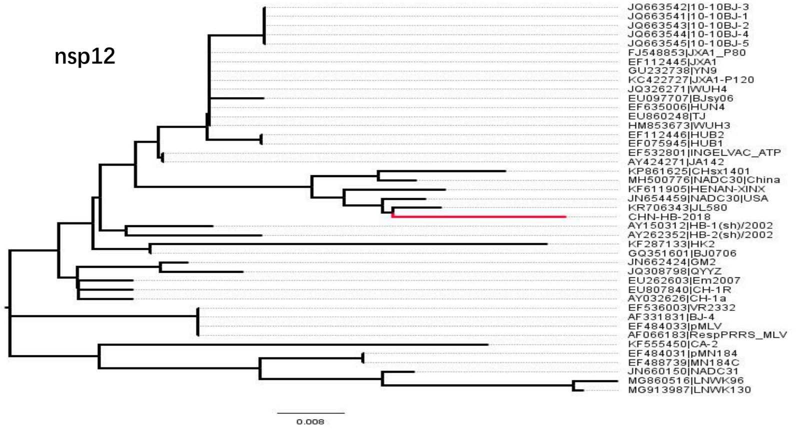
nsp12
JQ663542|10-10BJ-3
JQ663541|10-10BJ-1
JQ663543|10-10BJ-2
JQ663544|10-10BJ-4
JQ663545|10-10BJ-5
FJ548853|JXA1_P80
EF112445|JXA1
GU232738|YN9
KC422727|JXA1-P120
JQ326271|WUH4
EU097707|BJsy06
EF635006|HUN4
EU860248|TJ
HM853673|WUH3
EF112446|HUB2
EF075945|HUB1
EF532801|INGELVAC_ATP
AY424271|JA142
KP861625|CHsx1401
MH500776|NADC30|China
KF611905|HENAN-XINX
JN654459|NADC30|USA
KR706343|JL580
CHN-HB-2018
AY150312|HB-1(sh)/2002
AY262352|HB-2(sh)/2002
KF287133|HK2
GQ351601|BJ0706
JN662424|GM2
JQ308798|QYYZ
EU262603|Em2007
EU807840|CH-1R
AY032626|CH-1a
EF536003|VR2332
AF331831|BJ-4
EF484033|pMLV
AF066183|RespPRRS_MLV
KF555450|CA-2
EF484031|pMN184
EF488739|MN184C
JN660150|NADC31
MG860516|LNWK96
MG913987|LNWK130
0.008

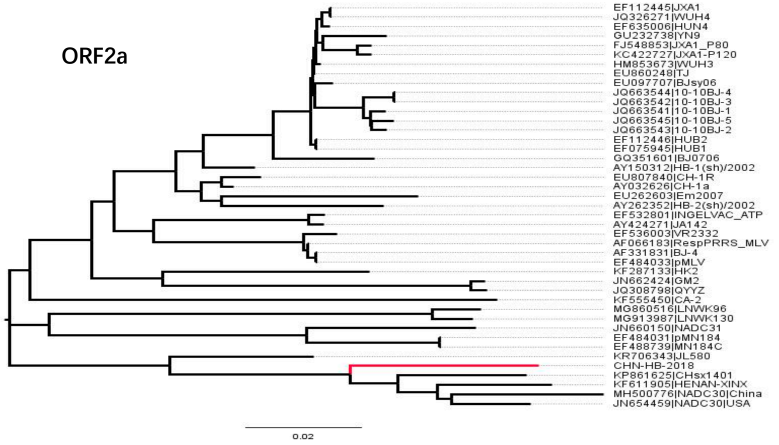
ORF2a
EF112445|JXA1
JQ326271|WUH4
EF635006|HUN4
GU232738|YN9
FJ548853|JXA1_P80
KC422727|JXA1-P120
HM853673|WUH3
EU860248|TJ
EU097707|BJsy06
JQ663544|10-10BJ-4
JQ663542|10-10BJ-3
JQ663541|10-10BJ-1
JQ663545|10-10BJ-5
JQ663543|10-10BJ-2
EF112446|HUB2
EF075945|HUB1
GQ351601|BJ0706
AY150312|HB-1(sh)/2002
EU807840|CH-1R
AY032626|CH-1a
EU262603|Em2007
AY262352|HB-2(sh)/2002
EF532801|INGELVAC_ATP
AY424271|JA142
EF536003|VR2332
AF066183|RespPRRS_MLV
AF331831|BJ-4
EF484033|pMLV
KF287133|HK2
JN662424|GM2
JQ308798|QYYZ
KF555450|CA-2
MG860516|LNWK96
MG913987|LNWK130
JN660150|NADC31
EF484031|pMN184
EF488739|MN184C
KR706343|JL580
CHN-HB-2018
KP861625|CHsx1401
KF611905|HENAN-XINX
MH500776|NADC30|China
JN654459|NADC30|USA
0.02

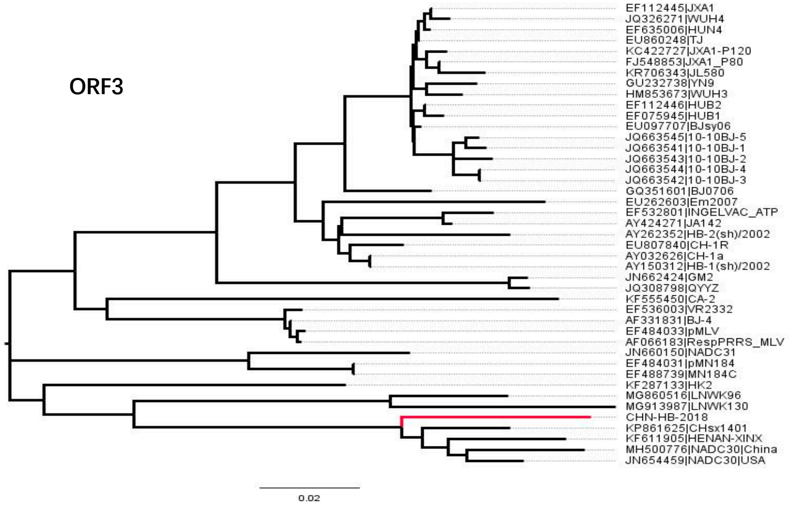
ORF3
EF112445|JXA1
JQ326271|WUH4
EF635006|HUN4
EU860248|TJ
KC422727|JXA1-P120
FJ548853|JXA1_P80
KR706343|JL580
GU232738|YN9
HM853673|WUH3
EF112446|HUB2
EF075945|HUB1
EU097707|BJsy06
JQ663545|10-10BJ-5
JQ663541|10-10BJ-1
JQ663543|10-10BJ-2
JQ663544|10-10BJ-4
JQ663542|10-10BJ-3
GQ351601|BJ0706
EU262603|Em2007
EF532801|INGELVAC_ATP
AY424271|JA142
AY262352|HB-2(sh)/2002
EU807840|CH-1R
AY032626|CH-1a
AY150312|HB-1(sh)/2002
JN662424|GM2
JQ308798|QYYZ
KF555450|CA-2
EF536003|VR2332
AF331831|BJ-4
EF484033|pMLV
AF066183|RespPRRS_MLV
JN660150|NADC31
EF484031|pMN184
EF488739|MN184C
KF287133|HK2
MG860516|LNWK96
MG913987|LNWK130
CHN-HB-2018
KP861625|CHsx1401
KF611905|HENAN-XINX
MH500776|NADC30|China
JN654459|NADC30|USA
0.02

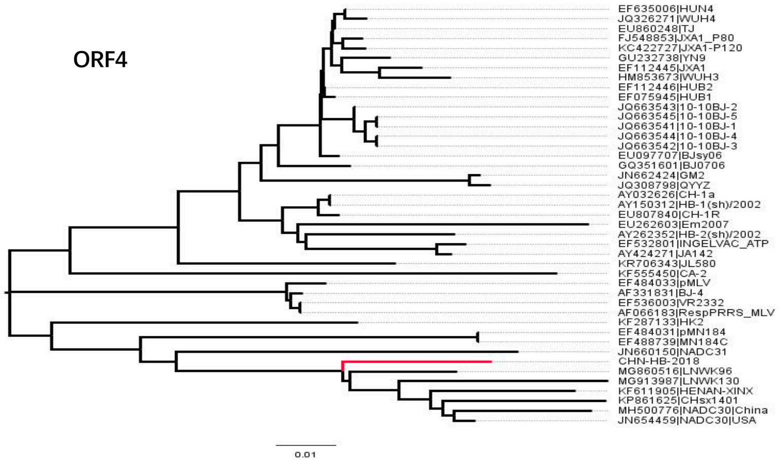
ORF4
EF635006|HUN4
JQ326271|WUH4
EU860248|TJ
FJ548853|JXA1_P80
KC422727|JXA1-P120
GU232738|YN9
EF112445|JXA1
HM853673|WUH3
EF112446|HUB2
EF075945|HUB1
JQ663543|10-10BJ-2
JQ663545|10-10BJ-5
JQ663541|10-10BJ-1
JQ663544|10-10BJ-4
JQ663542|10-10BJ-3
EU097707|BJsy06
GQ351601|BJ0706
JN662424|GM2
JQ308798|QYYZ
AY032626|CH-1a
AY150312|HB-1(sh)/2002
EU807840|CH-1R
EU262603|Em2007
AY262352|HB-2(sh)/2002
EF532801|INGELVAC_ATP
AY424271|JA142
KR706343|JL580
KF555450|CA-2
EF484033|pMLV
AF331831|BJ-4
EF536003|VR2332
AF066183|RespPRRS_MLV
KF287133|HK2
EF484031|pMN184
EF488739|MN184C
JN660150|NADC31
CHN-HB-2018
MG860516|LNWK96
MG913987|LNWK130
KF611905|HENAN-XINX
KP861625|CHsx1401
MH500776|NADC30|China
JN654459|NADC30|USA
0.01

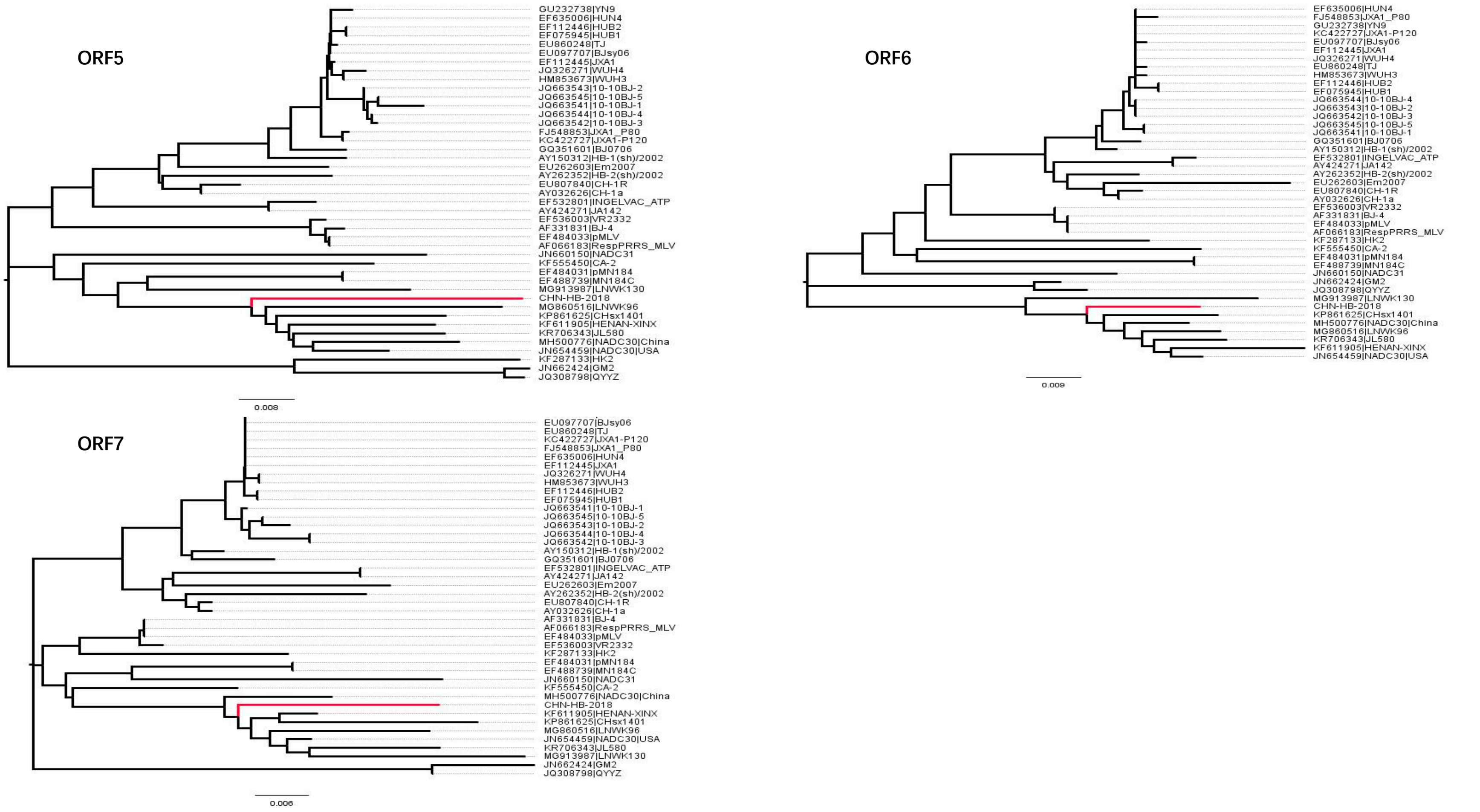
ORF5
GU232738|YN9
EF635006|HUN4
EF112446|HUB2
EF075945|HUB1
EU860248|TJ
EU097707|BJsy06
EF112445|JXA1
JQ326271|WUH4
HM853673|WUH3
JQ663543|10-10BJ-2
JQ663545|10-10BJ-5
JQ663541|10-10BJ-1
JQ663544|10-10BJ-4
JQ663542|10-10BJ-3
FJ548853|JXA1_P80
KC422727|JXA1-P120
GQ351601|BJ0706
AY150312|HB-1(sh)/2002
EU262603|Em2007
AY262352|HB-2(sh)/2002
EU807840|CH-1R
AY032626|CH-1a
EF532801|INGELVAC_ATP
AY424271|JA142
EF536003|VR2332
AF331831|BJ-4
EF484033|pMLV
AF066183|RespPRRS_MLV
JN660150|NADC31
KF555450|CA-2
EF484031|pMN184
EF488739|MN184C
MG913987|LNWK130
CHN-HB-2018
MG860516|LNWK96
KP861625|CHsx1401
KF611905|HENAN-XINX
KR706343|JL580
MH500776|NADC30|China
JN654459|NADC30|USA
KF287133|HK2
JN662424|GM2
JQ308798|QYYZ
0.008
ORF6
EF635006|HUN4
FJ548853|JXA1_P80
GU232738|YN9
KC422727|JXA1-P120
EU097707|BJsy06
EF112445|JXA1
JQ326271|WUH4
EU860248|TJ
HM853673|WUH3
EF112446|HUB2
EF075945|HUB1
JQ663544|10-10BJ-4
JQ663543|10-10BJ-2
JQ663542|10-10BJ-3
JQ663545|10-10BJ-5
JQ663541|10-10BJ-1
GQ351601|BJ0706
AY150312|HB-1(sh)/2002
EF532801|INGELVAC_ATP
AY424271|JA142
AY262352|HB-2(sh)/2002
EU262603|Em2007
EU807840|CH-1R
AY032626|CH-1a
EF536003|VR2332
AF331831|BJ-4
EF484033|pMLV
AF066183|RespPRRS_MLV
KF287133|HK2
KF555450|CA-2
EF484031|pMN184
EF488739|MN184C
JN660150|NADC31
JN662424|GM2
JQ308798|QYYZ
MG913987|LNWK130
CHN-HB-2018
KP861625|CHsx1401
MH500776|NADC30|China
MG860516|LNWK96
KR706343|JL580
KF611905|HENAN-XINX
JN654459|NADC30|USA
0.009
ORF7
EU097707|BJsy06
EU860248|TJ
KC422727|JXA1-P120
FJ548853|JXA1_P80
EF635006|HUN4
EF112445|JXA1
JQ326271|WUH4
HM853673|WUH3
EF112446|HUB2
EF075945|HUB1
JQ663541|10-10BJ-1
JQ663545|10-10BJ-5
JQ663543|10-10BJ-2
JQ663544|10-10BJ-4
JQ663542|10-10BJ-3
AY150312|HB-1(sh)/2002
GQ351601|BJ0706
EF532801|INGELVAC_ATP
AY424271|JA142
EU262603|Em2007
AY262352|HB-2(sh)/2002
EU807840|CH-1R
AY032626|CH-1a
AF331831|BJ-4
AF066183|RespPRRS_MLV
EF484033|pMLV
EF536003|VR2332
KF287133|HK2
EF484031|pMN184
EF488739|MN184C
JN660150|NADC31
KF555450|CA-2
MH500776|NADC30|China
CHN-HB-2018
KF611905|HENAN-XINX
KP861625|CHsx1401
MG860516|LNWK96
JN654459|NADC30|USA
KR706343|JL580
MG913987|LNWK130
JN662424|GM2
JQ308798|QYYZ
0.006

Supplementary Figure S2

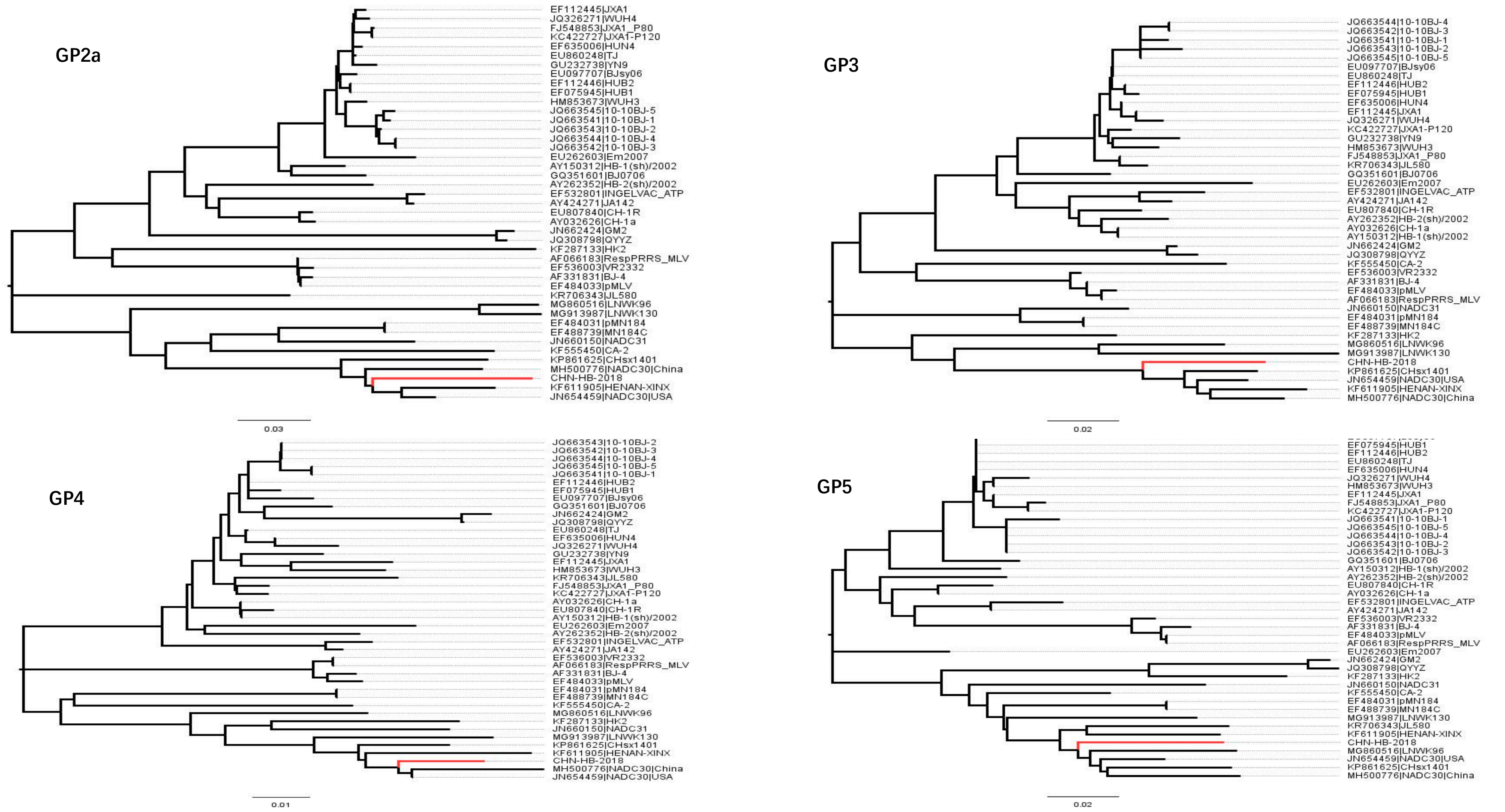

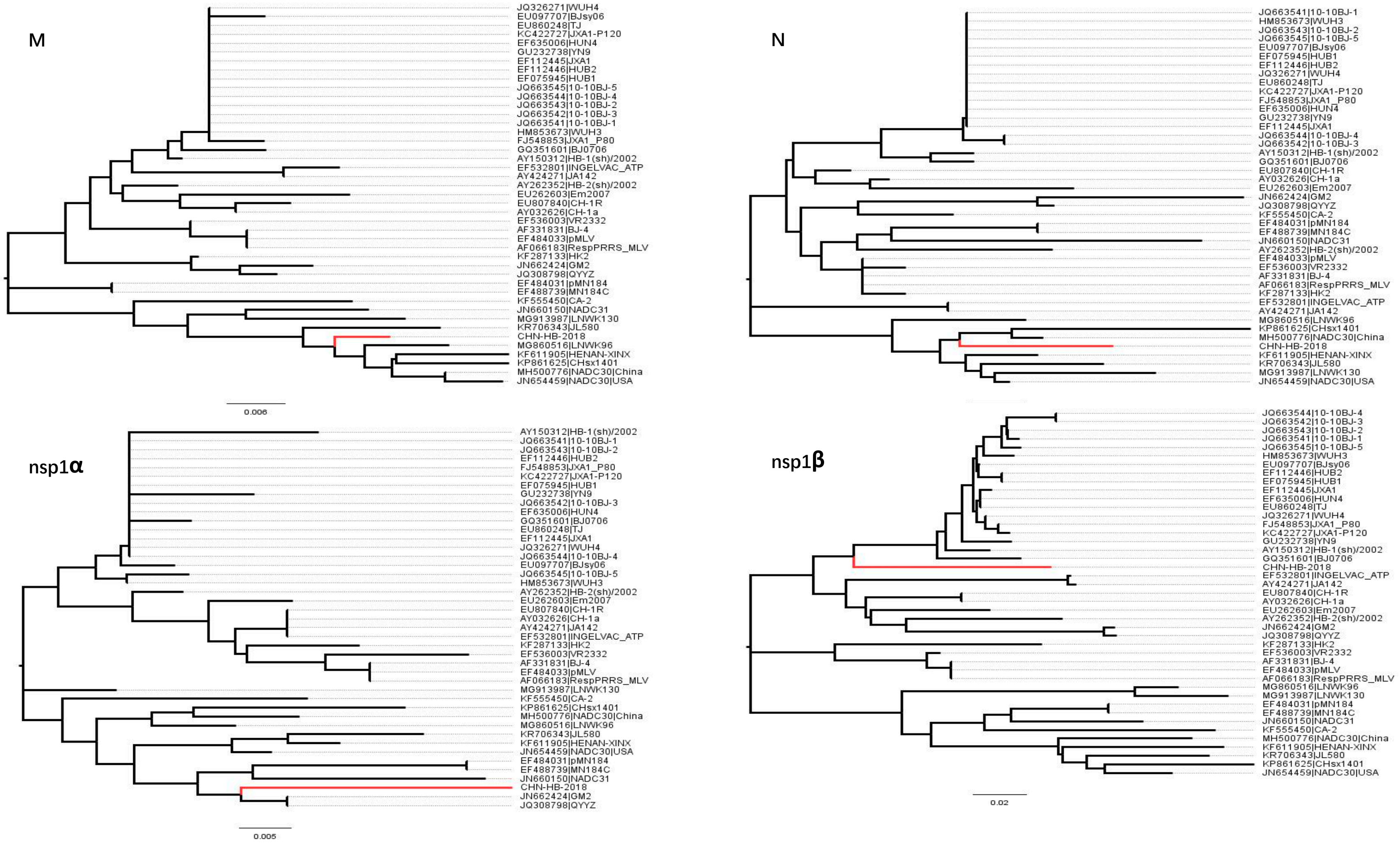
M
JQ326271|WUH4
EU097707|BJsy06
EU860248|TJ
KC422727|JXA1-P120
EF635006|HUN4
GU232738|YN9
EF112445|JXA1
EF112446|HUB2
EF075945|HUB1
JQ663545|10-10BJ-5
JQ663544|10-10BJ-4
JQ663543|10-10BJ-2
JQ663542|10-10BJ-3
JQ663541|10-10BJ-1
HM853673|WUH3
FJ548853|JXA1_P80
GQ351601|BJ0706
AY150312|HB-1(sh)/2002
EF532801|INGELVAC_ATP
AY424271|JA142
AY262352|HB-2(sh)/2002
EU262603|Em2007
EU807840|CH-1R
AY032626|CH-1a
EF536003|VR2332
AF331831|BJ-4
EF484033|pMLV
AF066183|RespPRRS_MLV
KF287133|HK2
JN662424|GM2
JQ308798|QYYZ
EF484031|pMN184
EF488739|MN184C
KF555450|CA-2
JN660150|NADC31
MG913987|LNWK130
KR706343|JL580
CHN-HB-2018
MG860516|LNWK96
KF611905|HENAN-XINX
KP861625|CHsx1401
MH500776|NADC30|China
JN654459|NADC30|USA
0.006
N
JQ663541|10-10BJ-1
HM853673|WUH3
JQ663543|10-10BJ-2
JQ663545|10-10BJ-5
EU097707|BJsy06
EF075945|HUB1
EF112446|HUB2
JQ326271|WUH4
EU860248|TJ
KC422727|JXA1-P120
FJ548853|JXA1_P80
EF635006|HUN4
GU232738|YN9
EF112445|JXA1
JQ663544|10-10BJ-4
JQ663542|10-10BJ-3
AY150312|HB-1(sh)/2002
GQ351601|BJ0706
EU807840|CH-1R
AY032626|CH-1a
EU262603|Em2007
JN662424|GM2
JQ308798|QYYZ
KF555450|CA-2
EF484031|pMN184
EF488739|MN184C
JN660150|NADC31
AY262352|HB-2(sh)/2002
EF484033|pMLV
EF536003|VR2332
AF331831|BJ-4
AF066183|RespPRRS_MLV
KF287133|HK2
EF532801|INGELVAC_ATP
AY424271|JA142
MG860516|LNWK96
KP861625|CHsx1401
MH500776|NADC30|China
CHN-HB-2018
KF611905|HENAN-XINX
KR706343|JL580
MG913987|LNWK130
JN654459|NADC30|USA
nsp1α
AY150312|HB-1(sh)/2002
JQ663541|10-10BJ-1
JQ663543|10-10BJ-2
EF112446|HUB2
FJ548853|JXA1_P80
KC422727|JXA1-P120
EF075945|HUB1
GU232738|YN9
JQ663542|10-10BJ-3
EF635006|HUN4
GQ351601|BJ0706
EU860248|TJ
EF112445|JXA1
JQ326271|WUH4
JQ663544|10-10BJ-4
EU097707|BJsy06
JQ663545|10-10BJ-5
HM853673|WUH3
AY262352|HB-2(sh)/2002
EU262603|Em2007
EU807840|CH-1R
AY032626|CH-1a
AY424271|JA142
EF532801|INGELVAC_ATP
KF287133|HK2
EF536003|VR2332
AF331831|BJ-4
EF484033|pMLV
AF066183|RespPRRS_MLV
MG913987|LNWK130
KF555450|CA-2
KP861625|CHsx1401
MH500776|NADC30|China
MG860516|LNWK96
KR706343|JL580
KF611905|HENAN-XINX
JN654459|NADC30|USA
EF484031|pMN184
EF488739|MN184C
JN660150|NADC31
CHN-HB-2018
JN662424|GM2
JQ308798|QYYZ
0.005
nsp1β
JQ663544|10-10BJ-4
JQ663542|10-10BJ-3
JQ663543|10-10BJ-2
JQ663541|10-10BJ-1
JQ663545|10-10BJ-5
HM853673|WUH3
EU097707|BJsy06
EF112446|HUB2
EF075945|HUB1
EF112445|JXA1
EF635006|HUN4
EU860248|TJ
JQ326271|WUH4
FJ548853|JXA1_P80
KC422727|JXA1-P120
GU232738|YN9
AY150312|HB-1(sh)/2002
GQ351601|BJ0706
CHN-HB-2018
EF532801|INGELVAC_ATP
AY424271|JA142
EU807840|CH-1R
AY032626|CH-1a
EU262603|Em2007
AY262352|HB-2(sh)/2002
JN662424|GM2
JQ308798|QYYZ
KF287133|HK2
EF536003|VR2332
AF331831|BJ-4
EF484033|pMLV
AF066183|RespPRRS_MLV
MG860516|LNWK96
MG913987|LNWK130
EF484031|pMN184
EF488739|MN184C
JN660150|NADC31
KF555450|CA-2
MH500776|NADC30|China
KF611905|HENAN-XINX
KR706343|JL580
KP861625|CHsx1401
JN654459|NADC30|USA
0.02

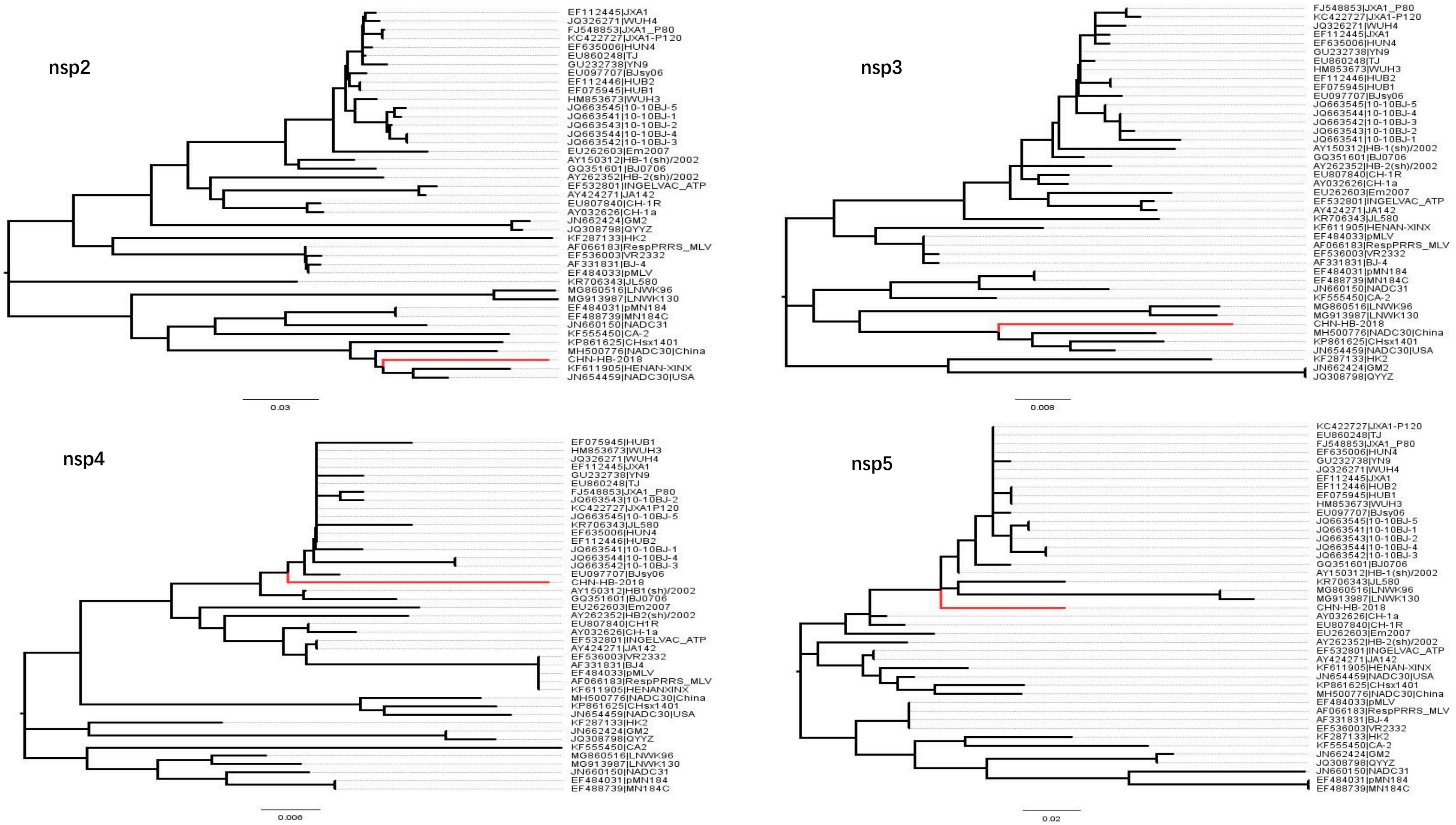
nsp2
0.03
nsp3
0.008
nsp4
0.006
nsp5
0.02

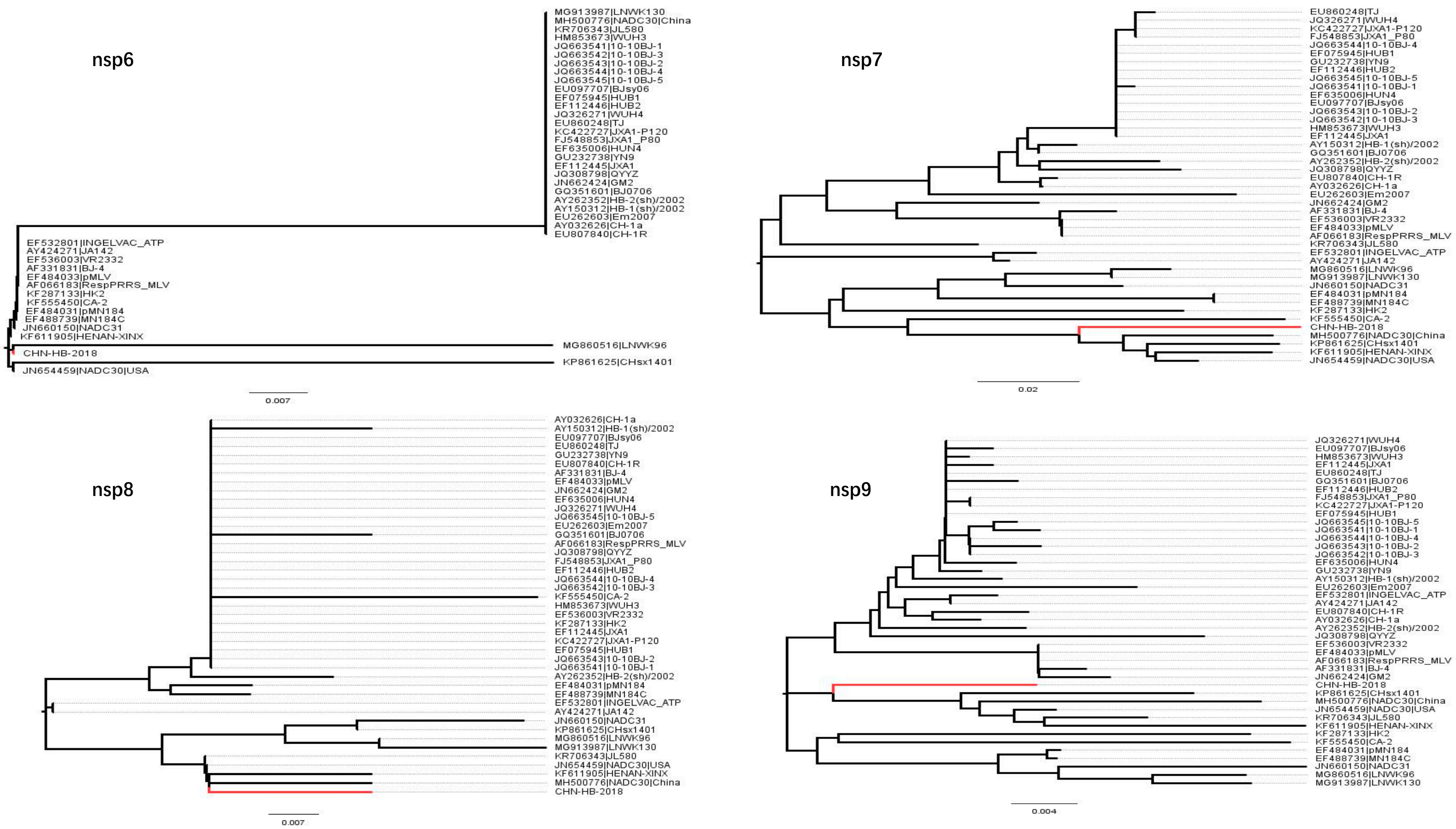
nsp6
MG913987|LNWK130
MH500776|NADC30|China
KR706343|JL580
HM853673|WUH3
JQ663541|10-10BJ-1
JQ663542|10-10BJ-3
JQ663543|10-10BJ-2
JQ663544|10-10BJ-4
JQ663545|10-10BJ-5
EU097707|BJsy06
EF075945|HUB1
EF112446|HUB2
JQ326271|WUH4
EU860248|TJ
KC422727|JXA1-P120
FJ548853|JXA1_P80
EF635006|HUN4
GU232738|YN9
EF112445|JXA1
JQ308798|QYYZ
JN662424|GM2
GQ351601|BJ0706
AY262352|HB-2(sh)/2002
AY150312|HB-1(sh)/2002
EU262603|Em2007
AY032626|CH-1a
EU807840|CH-1R
EF532801|INGELVAC_ATP
AY424271|JA142
EF536003|VR2332
AF331831|BJ-4
EF484033|pMLV
AF066183|RespPRRS_MLV
KF287133|HK2
KF555450|CA-2
EF484031|pMN184
EF488739|MN184C
JN660150|NADC31
KF611905|HENAN-XINX
MG860516|LNWK96
CHN-HB-2018
KP861625|CHsx1401
JN654459|NADC30|USA
0.007
nsp7
EU860248|TJ
JQ326271|WUH4
KC422727|JXA1-P120
FJ548853|JXA1_P80
JQ663544|10-10BJ-4
EF075945|HUB1
GU232738|YN9
EF112446|HUB2
JQ663545|10-10BJ-5
JQ663541|10-10BJ-1
EF635006|HUN4
EU097707|BJsy06
JQ663543|10-10BJ-2
JQ663542|10-10BJ-3
HM853673|WUH3
EF112445|JXA1
AY150312|HB-1(sh)/2002
GQ351601|BJ0706
AY262352|HB-2(sh)/2002
JQ308798|QYYZ
EU807840|CH-1R
AY032626|CH-1a
EU262603|Em2007
JN662424|GM2
AF331831|BJ-4
EF536003|VR2332
EF484033|pMLV
AF066183|RespPRRS_MLV
KR706343|JL580
EF532801|INGELVAC_ATP
AY424271|JA142
MG860516|LNWK96
MG913987|LNWK130
JN660150|NADC31
EF484031|pMN184
EF488739|MN184C
KF287133|HK2
KF555450|CA-2
CHN-HB-2018
MH500776|NADC30|China
KP861625|CHsx1401
KF611905|HENAN-XINX
JN654459|NADC30|USA
0.02
nsp8
AY032626|CH-1a
AY150312|HB-1(sh)/2002
EU097707|BJsy06
EU860248|TJ
GU232738|YN9
EU807840|CH-1R
AF331831|BJ-4
EF484033|pMLV
JN662424|GM2
EF635006|HUN4
JQ326271|WUH4
JQ663545|10-10BJ-5
EU262603|Em2007
GQ351601|BJ0706
AF066183|RespPRRS_MLV
JQ308798|QYYZ
FJ548853|JXA1_P80
EF112446|HUB2
JQ663544|10-10BJ-4
JQ663542|10-10BJ-3
KF555450|CA-2
HM853673|WUH3
EF536003|VR2332
KF287133|HK2
EF112445|JXA1
KC422727|JXA1-P120
EF075945|HUB1
JQ663543|10-10BJ-2
JQ663541|10-10BJ-1
AY262352|HB-2(sh)/2002
EF484031|pMN184
EF488739|MN184C
EF532801|INGELVAC_ATP
AY424271|JA142
JN660150|NADC31
KP861625|CHsx1401
MG860516|LNWK96
MG913987|LNWK130
KR706343|JL580
JN654459|NADC30|USA
KF611905|HENAN-XINX
MH500776|NADC30|China
CHN-HB-2018
0.007
nsp9
JQ326271|WUH4
EU097707|BJsy06
HM853673|WUH3
EF112445|JXA1
EU860248|TJ
GQ351601|BJ0706
EF112446|HUB2
FJ548853|JXA1_P80
KC422727|JXA1-P120
EF075945|HUB1
JQ663545|10-10BJ-5
JQ663541|10-10BJ-1
JQ663544|10-10BJ-4
JQ663543|10-10BJ-2
JQ663542|10-10BJ-3
EF635006|HUN4
GU232738|YN9
AY150312|HB-1(sh)/2002
EU262603|Em2007
EF532801|INGELVAC_ATP
AY424271|JA142
EU807840|CH-1R
AY032626|CH-1a
AY262352|HB-2(sh)/2002
JQ308798|QYYZ
EF536003|VR2332
EF484033|pMLV
AF066183|RespPRRS_MLV
AF331831|BJ-4
JN662424|GM2
CHN-HB-2018
KP861625|CHsx1401
MH500776|NADC30|China
JN654459|NADC30|USA
KR706343|JL580
KF611905|HENAN-XINX
KF287133|HK2
KF555450|CA-2
EF484031|pMN184
EF488739|MN184C
JN660150|NADC31
MG860516|LNWK96
MG913987|LNWK130
0.004

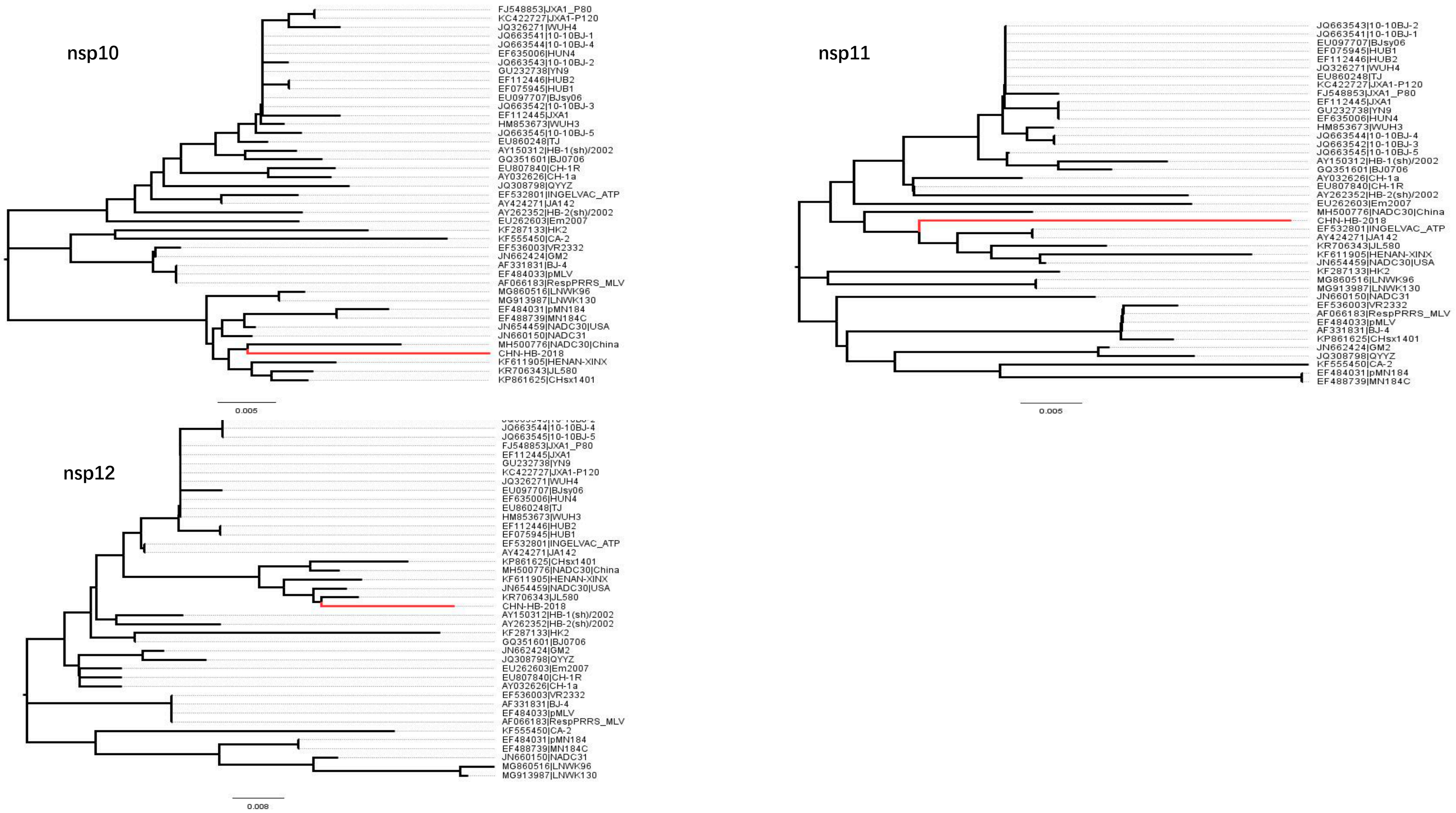
nsp10
FJ548853|JXA1_P80
KC422727|JXA1-P120
JQ326271|WUH4
JQ663541|10-10BJ-1
JQ663544|10-10BJ-4
EF635006|HUN4
JQ663543|10-10BJ-2
GU232738|YN9
EF112446|HUB2
EF075945|HUB1
EU097707|BJsy06
JQ663542|10-10BJ-3
EF112445|JXA1
HM853673|WUH3
JQ663545|10-10BJ-5
EU860248|TJ
AY150312|HB-1(sh)/2002
GQ351601|BJ0706
EU807840|CH-1R
AY032626|CH-1a
JQ308798|QYYZ
EF532801|INGELVAC_ATP
AY424271|JA142
AY262352|HB-2(sh)/2002
EU262603|Em2007
KF287133|HK2
KF555450|CA-2
EF536003|VR2332
JN662424|GM2
AF331831|BJ-4
EF484033|pMLV
AF066183|RespPRRS_MLV
MG860516|LNWK96
MG913987|LNWK130
EF484031|pMN184
EF488739|MN184C
JN654459|NADC30|USA
JN660150|NADC31
MH500776|NADC30|China
CHN-HB-2018
KF611905|HENAN-XINX
KR706343|JL580
KP861625|CHsx1401
0.005
nsp11
JQ663543|10-10BJ-2
JQ663541|10-10BJ-1
EU097707|BJsy06
EF075945|HUB1
EF112446|HUB2
JQ326271|WUH4
EU860248|TJ
KC422727|JXA1-P120
FJ548853|JXA1_P80
EF112445|JXA1
GU232738|YN9
EF635006|HUN4
HM853673|WUH3
JQ663544|10-10BJ-4
JQ663542|10-10BJ-3
JQ663545|10-10BJ-5
AY150312|HB-1(sh)/2002
GQ351601|BJ0706
AY032626|CH-1a
EU807840|CH-1R
AY262352|HB-2(sh)/2002
EU262603|Em2007
MH500776|NADC30|China
CHN-HB-2018
EF532801|INGELVAC_ATP
AY424271|JA142
KR706343|JL580
KF611905|HENAN-XINX
JN654459|NADC30|USA
KF287133|HK2
MG860516|LNWK96
MG913987|LNWK130
JN660150|NADC31
EF536003|VR2332
AF066183|RespPRRS_MLV
EF484033|pMLV
AF331831|BJ-4
KP861625|CHsx1401
JN662424|GM2
JQ308798|QYYZ
KF555450|CA-2
EF484031|pMN184
EF488739|MN184C
0.005
nsp12
JQ663544|10-10BJ-4
JQ663545|10-10BJ-5
FJ548853|JXA1_P80
EF112445|JXA1
GU232738|YN9
KC422727|JXA1-P120
JQ326271|WUH4
EU097707|BJsy06
EF635006|HUN4
EU860248|TJ
HM853673|WUH3
EF112446|HUB2
EF075945|HUB1
EF532801|INGELVAC_ATP
AY424271|JA142
KP861625|CHsx1401
MH500776|NADC30|China
KF611905|HENAN-XINX
JN654459|NADC30|USA
KR706343|JL580
CHN-HB-2018
AY150312|HB-1(sh)/2002
AY262352|HB-2(sh)/2002
KF287133|HK2
GQ351601|BJ0706
JN662424|GM2
JQ308798|QYYZ
EU262603|Em2007
EU807840|CH-1R
AY032626|CH-1a
EF536003|VR2332
AF331831|BJ-4
EF484033|pMLV
AF066183|RespPRRS_MLV
KF555450|CA-2
EF484031|pMN184
EF488739|MN184C
JN660150|NADC31
MG860516|LNWK96
MG913987|LNWK130
0.008
